# Native and engineered extracellular vesicles for wound healing

**DOI:** 10.3389/fbioe.2022.1053217

**Published:** 2022-12-09

**Authors:** Shengli Lu, Liping Lu, Yang Liu, Zenan Li, Yuan Fang, Zhizhao Chen, Jianda Zhou

**Affiliations:** ^1^ Department of Plastic Surgery, The Third Xiangya Hospital, Central South University, Changsha, China; ^2^ Department of Pediatrics, Nanfang Hospital, Southern Medical University, Guangzhou, China; ^3^ Department of Dermatology, Leiden University Medical Center, Leiden, Netherland

**Keywords:** wound healing, extracellular vesicles, exosome, engineered extracellular vesicles, chronic wound

## Abstract

Extracellular vesicles (EVs) that act as messengers mediate communication between parent and recipient cells through their contents, including nucleic acids, proteins, and lipids. These endogenous vesicles have emerged as a novel cell-free strategy for the treatment of diseases. EVs can be released by various types of cells with unique biological properties. Recent studies have shown that native EVs are used as therapeutic agents to promote tissue repair by delivering various growth factors and trophic factors including VEGF, EGF, TFN-α, IL-1β, and TGF-β to participate in all physiological processes of wound healing. Furthermore, to improve their specificity, safety, and efficiency for wound healing, the content and surface of EVs can be designed, modified, and engineered. The engineering strategies of EVs are divided into parent cell modification and indirect modification of EVs. The therapeutic potential of current EVs and engineered EVs for wound healing still requires the exploration of their large-scale clinical applications through innovative approaches. Herein, we provide an overview of the current biological knowledge about wound healing and EVs, as well as the application of native EVs in promoting wound healing. We also outline recent advances in engineering EV methodologies to achieve ideal therapeutic potential. Finally, the therapeutic applications of engineered EVs in wound healing are reviewed, and the challenges and prospects for the translation of engineered EVs to clinical applications are discussed.

## 1 Introduction

The skin is the largest organ of the human body. As a barrier, it protects the human body from being invaded and damaged by pathogens and external adverse environmental changes. However, wounds tend to be inevitable when the skin suffers acute and chronic injuries, including trauma, burns, and metabolic and vascular diseases. These chronic wounds, such as large-area burns and diabetic wound ulcers, *etc.*, are difficult to heal, and may also be accompanied by various intractable complications. This clinical problem causes patients to suffer physically and psychologically. It also imposes a heavy burden on society and the economy (Rani and Ritter, 2016; Rousselle et al., 2019). Studies have shown that the cost of treatment for diabetic ulcers is about $50,000 per patient, and the healthcare system needs to pay more than $25 billion a year to treat chronic wounds, with the growing prevalence of diabetes mellitus and other chronic diseases (Han and Ceilley, 2017). In recent years, therapeutics using cells and cell products, especially extracellular vesicles (EVs), and natural nano-sized particles, have attracted intense attention in the field of research to promote wound healing (Rodriguez-Menocal et al., 2015; Rani and Ritter, 2016; An et al., 2021).

The EVs were first revealed after the observation of particles from procoagulant platelets in normal blood plasma (Chargaff and West, 1946). A growing number of studies describe the properties and functions of the natural nano-sized EVs derived from cells. All kinds of normal or tumor cells can release EVs which are enriched in biologically active cargos, including proteins, nucleic acids, lipids, and metabolites for intercellular communication and pharmacological effects (Zhang et al., 2019a). EVs are mainly divided into two subgroups, exosomes (30–100-nm) and microvesicles (MVs) (50–1,000-nm) according to their size and biogenesis mechanism (van Niel et al., 2018). Some specific cell-derived EVs have been used as therapeutic nanoparticles in various biomedical fields including drug delivery, immunotherapy, and regenerative medicine (Hao et al., 2021). The advantages of EVs lie in their excellent biocompatibility, low immunogenicity, negligible toxicity, and low cost. Moreover, accumulating evidence suggests that EVs can deliver growth factors, cytokines, and trophic factors (e.g., VEGFA, EGF, PDGFA, TNF-α, IL-1β, and MCP-1) to recipient injured cells, or induce recipient cells to release these factors, thus promisingly promoting wound repair processes by mediating various biological events, such as cell proliferation, anti-inflammation, angiogenesis and re-epithelization (Hu et al., 2018; Yang et al., 2020a; Wei et al., 2020; An et al., 2021). Therefore, the use of native EVs derived from different cells to accelerate wound healing is a promising area of research.

Although natural EVs have therapeutic potential for wound healing, some detrimental properties, such as low concentration, short half-life, rapid degradation in the wound area, and low-efficiency payload limit their further clinical applications. As a result, engineered EVs have recently been explored as new EV-based nanotherapeutics to overcome the limitations of natural EVs (Vader et al., 2016). Some engineering methods of EVs have been applied to wound repair with promising prospects, such as modification of EV membrane, incorporation of EV cargo, as well as the combination of EVs with other nanomaterials.

In our review, we believe that the engineered EVs will enrich the means of wound therapy with promising therapeutic potential. To begin with, we summarized the current knowledge on wound healing and EVs. Then, we discussed the therapeutic potential of native EVs from different sources during wound healing. We also described the recent advancements in EVs engineering methodologies. Finally, we outlined the emerging nanotherapeutics of engineered EVs for the treatment of wound healing and discussed the concerns and perspectives in the field.

## 2 The biology and mechanisms of wound healing

Wound healing is an extremely complex process that depends on the interaction between many regulated events, including coagulation, re-epithelialization, granulation tissue formation, and neovascularization. This dynamic and biological process consists of several overlapping stages which are divided into four interactive phases: hemostasis, inflammation, proliferation, and remodeling (Martin, 1997; Singer and Clark, 1999; Guillamat-Prats, 2021).

Hemostasis first occurs after wound initiation. After the release of thromboxane and prostaglandins, blood vessels immediately constrict in response to injury. Simultaneously, collagen, platelet, thrombin, and fibronectin are involved in the formation of blood dots to prevent blood loss and protect the wound from external microorganisms. Platelets also release cytokines, hormones, and chemokines for activating the healing stages (Furie and Furie, 2008). Then, various immune cells (e.g., monocytes, neutrophils, and lymphocytes) take part in the second stage of removing pathogens, apoptotic cells, and cell debris involved in wound healing from the wound bed (Furie and Furie, 2008). Neutrophils arrive at the wound first and clear pathogens and damaged cells by phagocytosis and secretion of proteases. Apoptotic bodies and cellular debris are eliminated by macrophages derived from local monocytes. Macrophages and the remaining neutrophils secrete a lot of growth factors that trigger the process of cell proliferation (Hart, 2002). During the proliferation stage, re-epithelialization of the injured tissue occurs owing to the proliferation of keratinocytes and fibroblasts from the wound edge (Velnar et al., 2009).

Angiogenesis is stimulated by an increase in growth factors including VEGF and FGF. Moreover, fibroblasts produce a new extracellular matrix (ECM) by secreting type III collagen, which contributes to wound closure (Velnar et al., 2009). Finally, type III collagen is gradually replaced by type I collagen during the remodeling phase. This is a longer process than the others. Adjustment of ECM structure and collagen breakdown lead to reduced wound size and thickness (Baum and Arpey, 2005; Velnar et al., 2009; Gantwerker and Hom, 2011).

## 3 Biogenesis, cargos, and uptake of EVs

**FIGURE 1:**
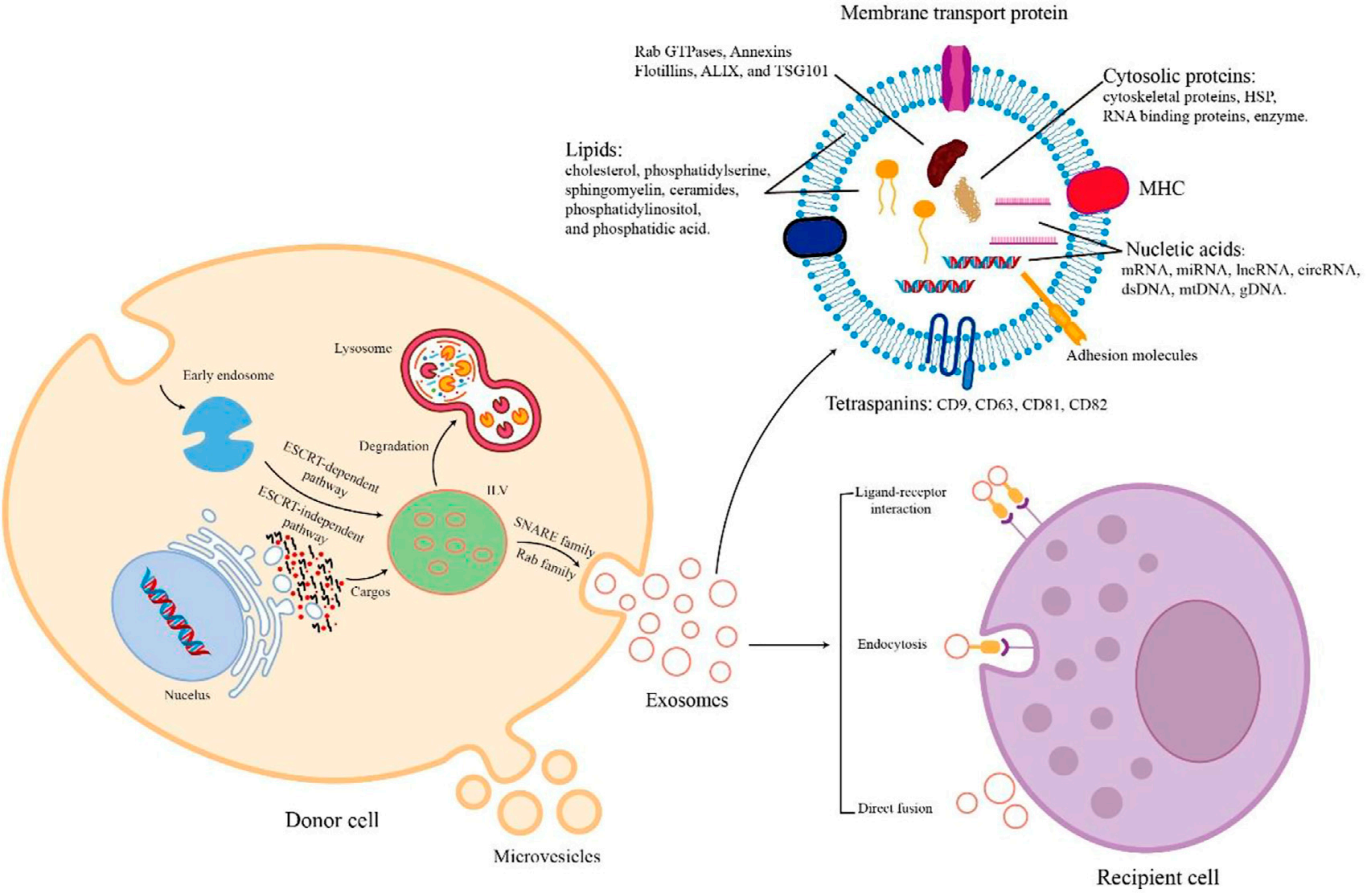
The biogenesis, cargos, and uptake of EVs. During EV biogenesis, early endosomes are formed after the inward budding of the plasma membranes and mature into late endosomes and MVBs containing ILVs. Exosomes are released by the fusion of MVBs and plasma membranes. Microvesicles are secreted through the direct outward budding of plasma membranes. EVs are rich in various cargos, such as proteins, nucleic acids, metabolites, and lipids. Recipient cells uptake EVs from donor cells through several mechanisms including ligand-receptor interaction, endocytosis, and direct fusion.

The process of EV biogenesis is different between exosomes and microvesicles. Exosomes originate from the endosomal system and are released through the fusion process of the plasma membrane with multivesicular bodies (MVBs) containing intraluminal vesicles (ILVs). In contrast, microvesicles are secreted through the process of outward budding of the cell membrane (Raposo and Stoorvogel, 2013). Although these two EVs originate from different cellular locations, their biogenesis mechanisms are similar (van Niel et al., 2018). There are mainly several stages in the biogenesis of exosomes Figure 1. Early endosomes are generated due to the inward budding of the plasma membrane. Then, they form into late endosomes and mature into MVBs containing ILVs. Next, MVB follows two different pathways. MVB is degraded by fusion with lysosomes. On the other hand, exosomes are released after MVB is transported to the cell membrane and fused with the cell membrane (Kahlert and Kalluri, 2013; Robbins and Morelli, 2014; van Niel et al., 2018). Exosomes are formed in MVBs through ESCRT-dependent or independent mechanisms (Raiborg and Stenmark, 2009). After MVBs are produced, the secretory MVBs fuse with the plasma membrane to release exosomes outside the cell. In recent years, the process of exosome release has gradually been clarified. It mainly depends on the auxiliary role of Rab and SNARE families (Abels and Breakefield, 2016).

During the biogenesis of EVs, various contents with biological activity are enriched in EVs. The cargos of EVs vary according to the way of biogenesis, cell type, and physiological conditions. All EVs commonly contain a series of nucleic acids, proteins, and lipids (Abels and Breakefield, 2016, Figure 1). EVs contain a variety of nucleic acids, including mRNA, miRNA, other non-coding RNA, double-stranded DNA, and mtDNA (Gusachenko et al., 2013). Certain proteins of EV are involved in EV biogenesis, such as Rab GTPases, Annexins, Flotillins, ALIX, and TSG101 (Géminard et al., 2004; Ruiz-Martinez et al., 2016). EVs also contain HSP70, HSP90, adhesion molecules, skeleton proteins, MHC-II, MHC-I, CD86, and tetraspanins (e.g., CD9, CD63, CD81, and CD82) (Pegtel and Gould, 2019; Müller, 2020). In addition, the lipids of EVs consist of cholesterol, ceramides, sphingomyelin, phosphatidylserine, phosphatidylinositol, and phosphatidic acid (Skotland et al., 2019). When extracellular vesicles are transported to recipient cells, their contents are released to perform various biological functions.

The uptake mechanism of EVs differs depending on their donor and recipient cells Figure 1. The process of direct fusion of the EV membrane and the cell membrane requires the participation of several protein families, such as SNAREs, Rab proteins, and SM-proteins (Jahn and Südhof, 1999). Endocytosis plays an important role in EV uptake. There are diverse endocytic pathways in cells, including clathrin-mediated pathways, caveolin-dependent uptake, macropinocytosis, phagocytosis, and lipid raft-associated internalization (Kirchhausen, 2000; Nabi and Le, 2003; Doherty and McMahon, 2009). Because of the presence of cell adhesion molecules (e.g., PS receptors, lectins, glycans, and integrins), EVs can bind with and be taken up by recipient cells to release their cargos by ligand-receptor interaction (van Dongen et al., 2016). Therefore, secreted EVs are taken up by recipient cells through these different mechanisms of cell-to-cell communication.

## 4 Native EVs for wound healing

**FIGURE 2:**
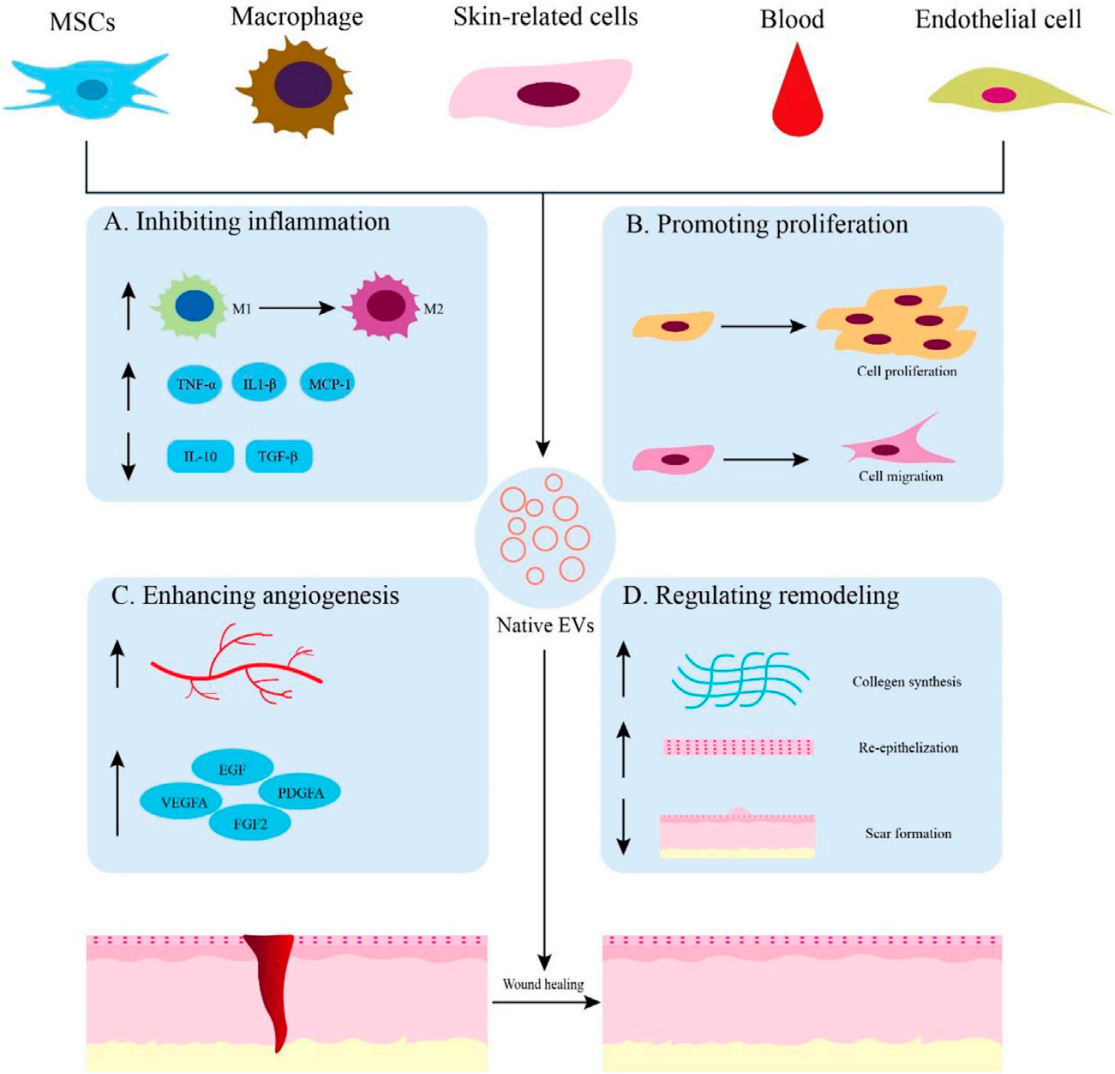
Native EVs are derived from different sources for wound healing. **(A)** Application of native EVs to inhibit inflammation, such as promoting macrophage polarization toward M2, increasing the expression of anti-inflammatory cytokines, and decreasing the expression of proinflammatory cytokines. **(B)** Functions of native EVs to promote cell proliferation and migration. **(C)** Effects of native EVs to enhance angiogenesis and up-regulate the expression of growth factors. **(D)** Using native EVs to regulate remodeling by promoting collagen synthesis, re-epithelization, and scarless wound healing.

An increasing number of studies have focused on the effects of extracellular vesicles on wound healing. These EVs are released from a wide range of sources, including various stem cells, immune cells, skin cells, epithelial cells, and blood. Therefore, we reviewed the potential therapeutic effects of EVs derived from several major sources in the wound healing process Figure 2 and Table 1.

**TABLE 1:** The examples of native EVs for wound healing.

EV origin	Model	Disease	Route	Dosage of EVs	Effector molecules; pathways	Effects	Ref
UCMSCs	*In vitro In vivo* (Rat)	Diabetic skin wound	Peri-wound injection	*In vitro*: 20 μg/ml *In vivo*: 60 μg per animal	MiR-let-7b; let-7b/TLR4, TLR4/NF-κB/STAT3/AKT pathways	*In vitro*: ↑M2 macrophages ↓M1 macrophage *In vivo*: ↓M1 macrophages infiltration ↑new small capillaries ↑wound healing	Ti et al. (2015)
UCMSCs	*In vitro In vivo* (Rat)	Third-degree burn	Tail vein injection	*In vitro*: 1,000 μg/ml *In vivo*: 800 μg per animal	MiR-181c; TLR4/NF-κB/p65 pathway	*In vitro*: ↓total white blood cells *In vivo*: ↓total white blood cells ↓neutrophils and macrophages ↓inflammation	Li et al. (2016)
EPCs	*In vitro In vivo* (Rat)	Diabetic skin wound	Subcutaneous injection	*In vitro*: 2 × 10^10^ or 1 × 10^11^ particles/ml *In vivo*: 2 × 10^10^ or 1 × 10^11^ particles per animal	Not studied; Erk1/2 pathway	*In vitro*: **↑**ECs angiogenesis **↑**ECs proliferation and migration *In vivo*: ↑re-epithelialization ↓scar formation ↑collagen maturity ↑new blood vessels formation	Zhang et al. (2016a)
HiPSC-MSCs	*In vitro In vivo* (Rat)	Full-thickness skin wound	Subcutaneous injection	*In vitro*: 25–200 μg/ml *In vivo*: 200 μg per animal	Type I, III collagen and elastin; not studied	*In vitro*: ↑HUVECs angiogenesis ↑fibroblasts collagen and elastin secretion ↑proliferation and migration of fibroblasts and HUVECs *In vivo*: ↑re-epithelialization ↓scar widths ↑collagen maturity ↑new blood vessels formation and maturation	Zhang et al. (2015a)
ADMSCs	*In vitro In vivo* (mouse)	Full-thickness skin wound	Intravenous injection or local injection	*In vitro*: 50–100 μg/ml *In vivo*: 200 μg per animal	N-cadherin, cyclin-1, PCNA and collagen I, III; not studied	*In vitro*: ↑fibroblasts migration and proliferation ↑collagen synthesis *In vivo*: ↑collagen I and III distributions ↓scar formation	Hu et al. (2016)
Macrophages (RAW 264.7)	*In vitro In vivo* (Rat)	Diabetic skin wound	Subcutaneous injection	*In vitro*: 50–500 μg/ml *In vivo*: 100–1,000 μg per animal	TNF-α, IL-6, phosphorylated AKT and VEGF; AKT/VEGF pathway	*In vitro*: ↑HUVECs migration and proliferation ↑HUVECs angiogenesis ↑Matrigel tube formation *In vivo*: ↓diabetic wound size ↓neutrophils/macrophages infiltration ↑collagen deposition ↑re-epithelialization ↑neovascularization	Li et al. (2019a)
Dermal fibroblasts	*In vitro In vivo* (Rat)	Diabetic skin wound	Subcutaneous injection	*In vitro*: Undefined *In vivo*: 2,000 μg per animal	IL-1β, IL-6, IL-10, VEGF-A, PCNA, Bcl-2, Bax; AKT/β-catenin pathway	*In vitro*: ↑HUVECs migration and proliferation ↑HUVECs tube formation ↓inflammatory reaction of HUVECs *In vivo*: ↑re-epithelialization ↑collagen deposition ↑skin cell proliferation ↑angiogenesis ↓inflammation	Han et al. (2021)
Fibroblasts (L929 cells)	*In vitro In vivo* (mouse)	Full-thickness skin wound	Apply on the wound bed (covered with Tegaderm Film)	*In vitro*: 10–50 μg/ml *In vivo*: 100 μg per animal	MMP1, MMP3, COL3A1, and VEGF; not studied	*In vitro*: ↑fibroblasts migration and proliferation ↑ECs migration and tube formation *In vivo*: ↑collagen formation and maturation ↑new blood vessels	Oh et al. (2021)
PRP	*In vitro In vivo* (Rat)	Diabetic skin wound	Apply on the wound bed (mixed with SAH)	*In vitro*: 5–50 μg/ml *In vivo*: Undefined	YAP; RhoA/YAP, PI3K/Akt and Erk1/2 pathway	*In vitro*: ↑fibroblasts migration and proliferation ↑HMEC angiogenesis and proliferation*In vivo*: ↑re-epithelialization ↑new blood vessels ↑collagen remodeling	Guo et al. (2017)
UCB	*In vitro In vivo* (mouse)	Full-thickness skin wound	Subcutaneous injection	*In vitro*: 10–100 μg/well *In vivo*: 100 μg per animal	MiR-21-3p, PTEN and SPRY1; PI3K/Akt and ERK1/2 pathways	*In vitro*: ↑fibroblasts migration and proliferation ↑ECs angiogenesis *In vivo*: ↑re-epithelialization ↑new blood vessels ↓scar widths	Hu et al. (2018)
Plasma ECs	*In vitro In vivo* (mouse)	Diabetic skin wound	Subcutaneous injection	*In vitro*: 5–50 μg/well *In vivo*: 5 μg per animal	YAP; PI3K/Akt/mTOR pathway	*In vitro*: ↑fibroblasts proliferation ↓fibroblasts senescence *In vivo*: ↑collagen deposition ↑microvascular density ↑macrophages infiltration ↓fibroblasts senescence	Wei et al. (2020)

UCMSCs, Human umbilical cord mesenchymal stem cells; EPCs, Human endothelial progenitor cells; HiPSC-MSCs, Human induced pluripotent stem cells-derived mesenchymal stem cells; ADMSCs, Adipose-derived mesenchymal stem cells; ECs, Endothelial cells; HUVECs, Human umbilical vein endothelial cells; PRP, Platelet-rich plasma; SAH, Sodium alginate hydrogel; HMEC, Human microvascular endothelial cell line; UCB, Human umbilical cord blood plasma.

### 4.1 MSC-derived EVs in wound healing

Mesenchymal stem cells (MSCs) can differentiate into various types of cells due to their pluripotent capacity. They are derived from a range of tissues and organs, including adipose tissue, bone marrow, umbilical cord, placenta, umbilical cord blood, *etc.* (Pittenger et al., 1999; Le Blanc and Mougiakakos, 2012). EVs obtained from MSCs can deliver transcription factors, anti-inflammatory factors, and growth factors to recipient cells for tissue repair and regeneration (Nawaz et al., 2016). Studies have shown that MSC-derived EVs play a positive regulatory role in all stages of wound healing owing to their functions, suggesting that these EVs can be potential therapeutic agents for wound healing.

#### 4.1.1 MSC-derived EVs promote wound healing by regulating inflammation

While inflammation is a normal phase of wound healing, excessive inflammation is detrimental (Eming et al., 2007). Macrophages play an important role in the inflammatory phase, with two phenotypes of pro-inflammatory M1 and anti-inflammatory M2 (Koh and DiPietro, 2011). EVs obtained from MSCs promote the polarization of macrophages toward M2, thereby achieving anti-inflammation effects (Lo Sicco et al., 2017). Let-7b of MSC-derived EVs triggers this polarization by inhibiting TLR4 signaling (Ti et al., 2015). In addition, MSC-derived EVs alleviate inflammation by regulating cytokines.

These EVs inhibit proinflammatory enzymes including iNOS and COX-2; down-regulate cytokines and chemokines such as TNF-α, IL-1β, and MCP-1. In contrast, anti-inflammatory cytokines such as IL-10 are up-regulated to promote wound healing (Yang et al., 2015a; Li et al., 2016a; Yu et al., 2016). MSC-Exosomes can convert activated T lymphocytes to T-regulatory phenotype, thereby accelerating wound healing *via* decreasing the production of interferon-γ and accumulation of M1 phenotype macrophages (Nosbaum et al., 2016; Monguió-Tortajada et al., 2017). It has been shown that miRNAs from MSC-EVs exert immunomodulatory effects in this phase. The high expression of miR-21, −146a, and −181c in hUCMSC-exosomes is associated with the regulation of inflammation and immune responses (Ti et al., 2016).

#### 4.1.2 MSC-derived EVs promote wound healing by regulating proliferation

Recent studies have demonstrated that EVs obtained from MSCs can promote angiogenesis, a critical step in wound healing. The hADSCs-derived exosomes promote angiogenesis by transferring miR-125a to endothelial cells. MiR-125a inhibits the expression of angiogenic inhibitor delta-like 4 (DLL4) and accelerates the formation of endothelial tip cells (Liang et al., 2016). Microvesicles from ADSCs are enriched in miR‐31, which can be transferred to endothelial cells to enhance angiogenesis by suppressing factor-inhibiting HIF-1 in HUVECs (Kang et al., 2016). MSC-derived EVs also stimulate the expression of intrinsic factors for improving wound healing. For instance, exosomes obtained from ADSCs can increase the expression of SIRT3 which could promote angiogenesis, reduce oxidative stress, and decrease mitochondrial functional impairment and the inflammatory response in endothelial cells, thereby enhancing diabetic wound healing (Zhang et al., 2022). ADSC-microvesicles can upregulate the expression of growth factors such as VEGFA, PDGFA, EGF, and FGF2, as well as proliferative markers such as cyclin D1, cyclin D2, cyclin A1, and cyclin A2, to promote angiogenesis by activating AKT and ERK signaling pathways (Ren et al., 2019). Exosomes obtained from endothelial progenitor cells (EPCs) are able to promote angiogenic activities of endothelial cells by activating the ERK1/2 signaling pathway and up-regulating the expression of angiogenesis-related molecules (e.g., FGF-1, VEGFA, VEGFR-2, ANG-1, E-selectin, CXCL-16, eNOS, and IL-8) to enhance cutaneous wound healing in diabetic mice (Zhang et al., 2016a; Li et al., 2016b). In addition, exosomes derived from hiPSC-MSCs are qualified to induce angiogenesis and promote collagen synthesis, which can be used as therapeutic drugs for wound healing (Zhang et al., 2015a).

Epithelial regeneration of human dermal fibroblasts and keratinocytes is crucial for the proliferative phase of wound healing (Velnar et al., 2009). ADSCs‐Exos are taken up by fibroblasts and promote cell migration and proliferation. The synthesis of N-cadherin, Cyclin-1, PCNA, and collagen I, III is also increased due to the presence of ADSCs‐Exos (Hu et al., 2016). The functional lncRNA MALAT1 of ADSC-Exos can enhance ischemic wound healing by stimulating human dermal fibroblast migration and angiogenesis (Cooper et al., 2018). BMSC-derived CD63^+^ exosomes act as a carrier to transport external Wnt3a to recipient cells, resulting in the migration and proliferation of HDFs and promoting wound healing (McBride et al., 2017). HUCMSC-Exos accelerate re-epithelialization and cell proliferation by activating Wnt4/β-catenin and AKT signaling pathways and increases the expression of CK19, PCNA, and collagen I (Zhang et al., 2015b). Moreover, hiPSC-MSC-exosomes promote the secretion and mRNA expression of type I and type III collagen and, elastin, thereby, stimulating the proliferation and migration of HDFs and enhancing skin regeneration (Zhang et al., 2015a).

#### 4.1.3 MSC-derived EVs promote wound healing by regulating remodeling

The appearance and function of tissues and organs are affected by hypertrophic scar, which is an urgent problem for clinicians to solve in the tissue remodeling stage of wound healing (Martin, 1997). Regulating EV-related activities has been shown to play an active role in the inflammatory response, tissue homeostasis, and repair by recruiting wound-healing factors, EVs also play a crucial role in wound healing by matrix remodeling (Nawaz et al., 2018). As previously mentioned, exosomes obtained from ADSCs promoted skin regeneration by increasing the synthesis of collagen I and III in the early stage of wound healing, while reducing scar formation by inhibiting the expression of collagen (Hu et al., 2016). ADSCs-EVs can be a therapeutic tool for scarless wound healing by hindering the differentiation of fibroblasts into myofibroblasts, changing the ratio of several cellular molecules, such as the ratio of collagen type III-to-collagen type I, MMP3-to-MMP1, and TGF-β3-to-TGF-β1 (Wang et al., 2017). Also, HUCMSC-exosomes reduce scarring by inhibiting the TGF-β/SMAD2 signaling pathway, which prevents fibroblast from differentiating into myofibroblast (Fang et al., 2016). Additionally, excessive collagen deposition and fibroblast expansion can be suppressed by the 14-3-3ζ protein contained in hUCMSC-exosomes. This protein enhances YAP phosphorylation by regulating the binding of YAP substrates and p-LATS kinase, thereby inhibiting the activation of the Wnt/β-catenin signaling pathway *via* modulating the Hippo-YAP pathway (Zhang et al., 2016b). HiPSC-MSC-exosomes can enhance collagen maturation and reduce scar width by increasing the mRNA expression and production of type I and III collagen as well as elastin (Zhang et al., 2015a). The application of a high concentration of EVs derived from hAEC reduces extracellular matrix deposition by promoting MMP-1 expression, resulting in scarless wound healing (Zhao et al., 2017). All these studies indicate that MSC-EVs play a crucial role in the remodeling phase and can be used as a treatment to reduce scar formation.

### 4.2 Macrophage-derived EVs for wound healing

Studies have shown that macrophages and their EVs contain various cytokines and miRNAs which play a key role in alleviating inflammation through a paracrine mechanism with recipient cells (Zhang et al., 2018). Exosomes obtained from macrophages can accelerate angiogenesis, re-epithelization, and decrease the levels of pro-inflammatory cytokines (TNF-α and IL-6) to promote diabetic wound healing (Li et al., 2019a). Moreover, macrophage-derived EVs are involved in the process of scar formation. The lncRNA-ASLNCS5088 of M2 macrophage exosomes is transferred to fibroblasts for inhibiting the function of miR-200c-3p, resulting in increasing the expression of GLS and α-SMA to regulate scar formation (Chen et al., 2019). These researches indicate that macrophage-derived EVs provide a novel therapeutic approach to wound healing.

### 4.3 Skin-related cell-derived EVs for wound healing

During wound healing, EVs derived from skin-related cells play an important role in ECM deposition, tissue remodeling, and wound contraction. For instance, exosomes released from autologous dermal fibroblasts (DF-Exos) can exert therapeutic effects on diabetic wound healing, since DF-Exos accelerate several biological events including re-epithelialization, cell proliferation, collagen deposition, angiogenesis, and inflammation inhibition by activating Akt/β-catenin pathway (Han et al., 2021). Fibroblast-derived (L929 cell line) EVs (L929-EVs) have also been shown to be effective in wound healing by up-regulating the expression of VEGF, promoting collagen synthesis and maturation, as well as inducing the formation of blood vessels and skin appendages (Oh et al., 2021). To promote wound healing, keratinocyte microvesicles (KC-MVs) can regulate the expression of various genes and activate multiple signaling pathways in dermal fibroblasts while human KC-MV miR-21 activates the function of fibroblast and enhances angiogenesis (Huang et al., 2015; Li et al., 2019b). EVs, secreted by human keratinocytes (HaCaT), promote the migration, proliferation, and activation of ERK1/2 and P38 of human keratinocytes and fibroblasts by activating the MAPKinase pathway to promote wound healing (Glady et al., 2021). These studies demonstrate that EVs derived from skin-related cells have a potent capacity in promoting wound healing and represent a novel approach to chronic wound therapy.

### 4.4 Blood-derived EVs for wound healing

Several studies have shown that EVs obtained from the blood can promote wound healing. Platelet-rich plasma (PRP) is widely used in the treatment of chronic wounds. PRP-derived exosomes can regulate the proliferation, and migration of fibroblasts and endothelial cells by activating YAP positively for inducing re-epithelialization and angiogenesis in chronic wounds (Guo et al., 2017). In addition, the PRP-derived exosomes contain the key mediator USP15, which promotes the proliferation, migration, and tissue repair activity of immortalized keratinocytes by inducing the deubiquitination of EIF4A1 (Xu et al., 2021). Human umbilical cord blood (UCB) plasma is also a major source of exosomes for wound healing. According to reports, UCB-derived exosomes can transfer miR-21-3p into fibroblasts and endothelial cells. MiR-21-3p promotes proliferation, migration, and angiogenic activities of fibroblasts and endothelial cells by suppressing phosphatase and tensin homolog (PTEN) and sprouty homolog 1 (SPRY1), thereby enhancing the regenerative abilities of these cells to accelerate cutaneous wound healing (Hu et al., 2018). Therefore, researchers can explore and develop blood-derived EVs as a therapeutic tool to wound healing.

### 4.5 Endothelial cell-derived EVs for wound healing

EVs secreted by endothelial cells (ED-EVs), due to their functional contents, regulate the endothelial cell activities including monocyte adhesion, cell migration, and angiogenesis (Goetzl et al., 2017). Plasma ED-EVs can increase microvascular density, collagen deposition, and macrophage infiltration by suppressing the YAP phosphorylation and activating the PI3K/Akt/mTOR pathway to promote wound healing (Wei et al., 2020). In contrast, endothelial cell-derived small extracellular vesicles (sEVs) containing miR-106b-5p can activate fibroblast autophagy and reduce collagen production by decreasing the expression of ERK1/2, resulting in delayed cutaneous wound healing (Zeng et al., 2019). Furthermore, as JMJD3 and RIPK3 are inhibited, EVs derived from human umbilical vein endothelial cells (HUVECs) restrain the wound healing process by reducing the angiogenesis and collagen I synthesis (Qi et al., 2021). As a result, regardless of whether endothelial cell-derived EVs promote or inhibit wound healing, they can be used as a new therapeutic means or target for wound treatment.

To conclude, over the past decade, all of these findings indicate that native EVs can promote cutaneous wound healing effectively. However, there is still a long way to go to overcome the limitations of native EVs, improvement of their therapeutic potential, and realization of their clinical applications. Therefore, researchers applied bioengineering methods to overcome limitations, and enhance the properties of native EVs to accelerate their clinical translation.

## 5 Engineering methodologies of EVs

**FIGURE 3:**
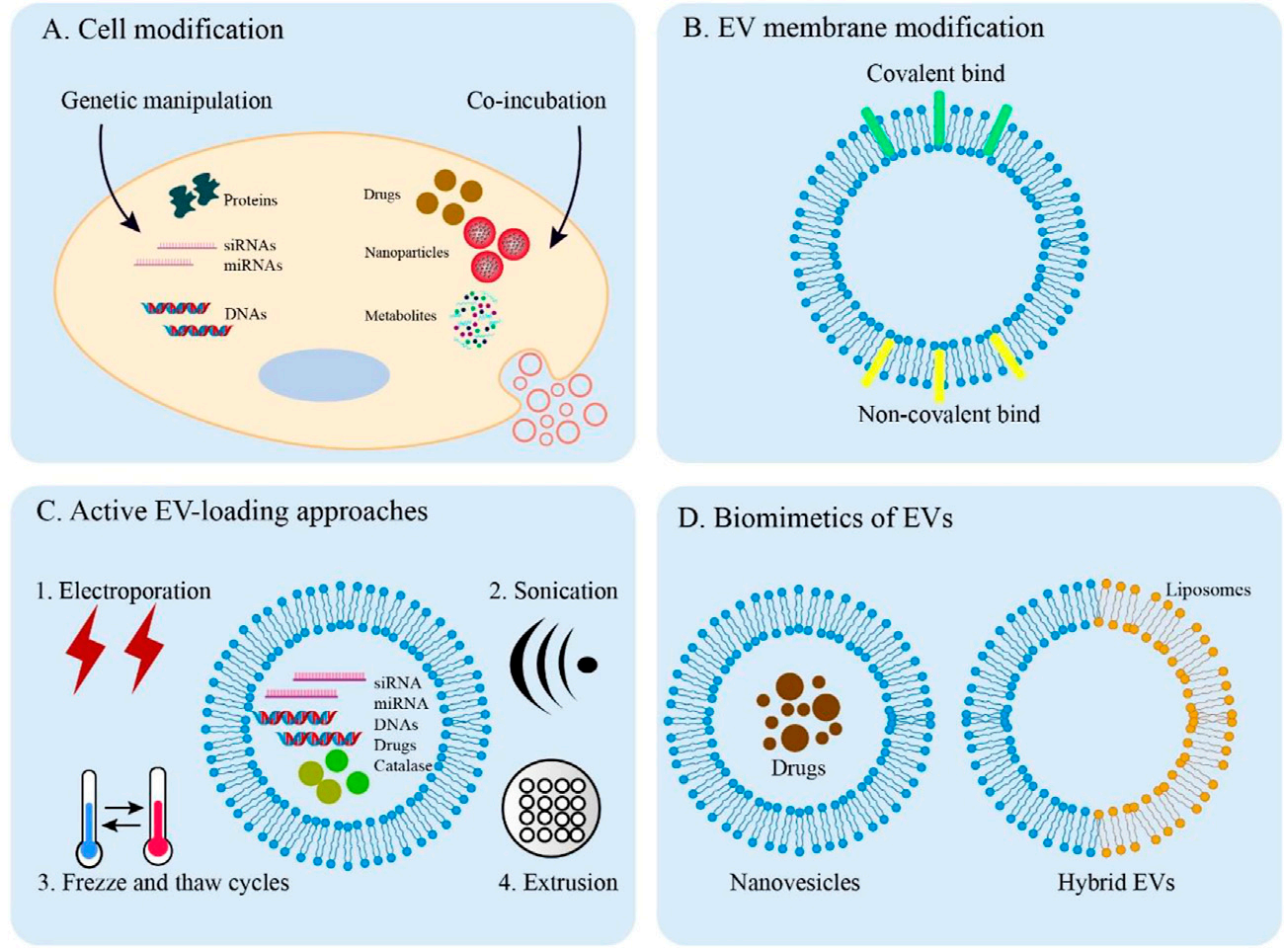
Overview of EV engineering approaches. **(A)** Direct methods for engineering donor cells. Engineered EVs can obtain desired cargos by genetic manipulation and co-incubation. **(B)** Covalent and non-covalent strategies for EV membrane modification. **(C)** Active methods are applied to load cargos into EVs through electroporation, sonication, freeze, and thaw cycles and extrusion. **(D)** The synthesized biomimetics of EVs (nanovesicles and hybrid EVs).

To better apply EVs by improving their function in clinical disease treatment, various engineering methodologies have been developed (Armstrong et al., 2017). Strategies for engineering EVs can be divided into two main types: indirect methods (cell modification) and direct methods (EV modification) Figure 3.

### 5.1 Indirect methodologies through cell modification

Cargo and surface protein expression of EVs can be altered by donor cell techniques, including genetic manipulation, co-incubation, and exogenous delivery. The biocompatibility, targeting and therapeutic abilities of EVs can be improved by these methods.

#### 5.1.1 Genetic manipulation

Genetic engineering is widely used in EVs modification for therapeutic applications. Gene transfection can produce engineered EVs by loading specific cargos into EVs and modifying EV membrane proteins. Exogenous nucleic acids (e.g., DNA, mRNA, siRNA, and miRNA) are transferred to parental cells, which increases the expression of nucleic acids through endogenous biogenesis for ideal EVs (Ridder et al., 2015). In addition, through the application of transgene expression, proteins that express on the EV membranes can be modified with targeting and homing characteristics (Mentkowski et al., 2018). Despite obtaining engineered EVs from this method being feasible and easy, the low loading efficiency and specificity still limit the better application in clinical medicine. Therefore, improvement of transfection efficiency and specificity should be further explored in future research.

#### 5.1.2 Co-incubation for cargo loading

Some drugs and drug-loaded small-sized nanoparticles can be packaged into engineered EVs by co-incubating them with parent cells of EVs. It has been shown that incubation of the MSCs with paclitaxel (PTX) can engineer EVs for robust anti-tumor activity (Pascucci et al., 2014). Doxorubicin and methotrexate were loaded into apoptotic bodies of tumor cells to apply the technique in cancer treatment (Tang et al., 2012). Leukemia can be treated by MSCs-derived charged EVs co-incubated with superparamagnetic iron oxide nanoparticles (Liu et al., 2017). Moreover, the culture medium of cells can be added to some unnatural metabolites, including lipids, amino acids, and glycans. Donor cells can uptake these molecules to regulate cellular biosynthesis. As a result, EVs obtained from these donor cells contain metabolically labeled sites such as endosomal proteins and cytoplasmic membrane lipids. For example, the EVs with azide-modified sialic acid can be released by binding tetraacetylated N-azidoacetyl-D-mannosamine to exosome-secreting cells (Wang et al., 2015). To raise loading efficiency, further studies on finding the appropriate drugs, and drug-loaded nanoparticles are needed.

### 5.2 Direct methodologies through EV modification

#### 5.2.1 EV membrane modification

With decreased clearance and desirable capabilities, the endowing of specific modifications of EV membranes can be covalent or non-covalent. The most common covalent method is to click chemistry which utilizes the reaction of copper-catalyzed azide-alkyne cyclo-addition. This approach rapidly forms chemical bonds and the molecules are directly covalently attached to the EV surface (Presolski et al., 2011). Neuropilin-1-targeted peptide and c (RGDyK) peptide are conjugated to EVs for the treatment of glioma and ischemic brain, respectively (Jia et al., 2018; Tian et al., 2018). PEGylation is a common method to modify the EV surfaces with polyethylene glycol, which can prolong the circulation half-life of the EVs (Kooijmans et al., 2016).

Additionally, non-covalent strategies are also involved in EV membrane modifications, such as electrostatic interactions, receptor-ligand binding, and hydrophobic insertion. The principle of the electrostatic method is that highly cationic species attach to negatively charged EV membrane functional groups (Susa et al., 2019). For example, cationic lipids bind to the EV surface *via* electrostatic interactions, which enhances the binding and uptake of positively charged EVs by recipient cells (Nakase and Futaki, 2015). Transferrin-bound superparamagnetic nanoparticle clusters are able to bind to the transferrin receptors present on the surface of exosomes to facilitate tumor targeting and therapy (Qi et al., 2016). Moreover, hydrophobic interactions can drive the integration of small lipophilic substances including common dyes into the EV membranes for EV membrane staining effectively (Harp et al., 2016). Through this method, various hydrophobic drugs such as curcumin, doxorubicin, paclitaxel, and methotrexate can be loaded into EVs for the treatment of inflammatory disease and cancer (Zhuang et al., 2011; Tang et al., 2012; Yang et al., 2015b; Smyth et al., 2015).

#### 5.2.2 Active EV-loading approaches

The active loading method allows efficient encapsulation of cargos using several techniques to transiently disrupt EV membranes, such as electroporation, sonication, freeze and thaw cycles, and extrusion. Electroporation facilitates the exogenous cargos including siRNAs, miRNAs, and drugs by applying an electrical field to transiently permeabilize the EV membranes (Cooper et al., 2014; Tian et al., 2014; Liang et al., 2018). Sonication enables drug diffusion by using a homogenizer probe to create pores in the EV membranes (Fuhrmann et al., 2015). Moreover, the freeze and thaw cycle and extrusion can also actively load cargos into EVs. For instance, it has been shown that catalase is loaded into exosomes by these two methods to treat Parkinson’s disease (Haney et al., 2015). However, all of these approaches have the potential to destabilize EV membranes, thereby affecting the integrity or therapeutic efficacy of EVs.

#### 5.2.3 Biomimetics of EVs

Some mimetics of EVs are synthesized to mimic the characteristics of natural EVs by using biomimetic molecules including EV proteins and lipids. These EV mimetics can serve as drug-delivery platforms for treating diseases. The doxorubicin-loaded EV-mimetic nanovesicles produced by the serial extrusion method enhance the ability of targeting and delivery of chemotherapeutic drugs for treating malignant tumors (Jang et al., 2013). Combining EVs with biomaterials can generate hybrid EVs, like hybrid exosome-liposome with advantages of both EVs and liposomes. For example, the phospholipid bilayer of artificial chimeric exosomes is used for anti-tumor therapy by combining cell membrane proteins from red blood cells and MCF-7 cancer cells (Zhang et al., 2019b). Because these biomimetics are similar to natural EVs so they have excellent biocompatibility and pharmacokinetics for clinical applications.

## 6 Engineered EVs for wound healing

**FIGURE 4:**
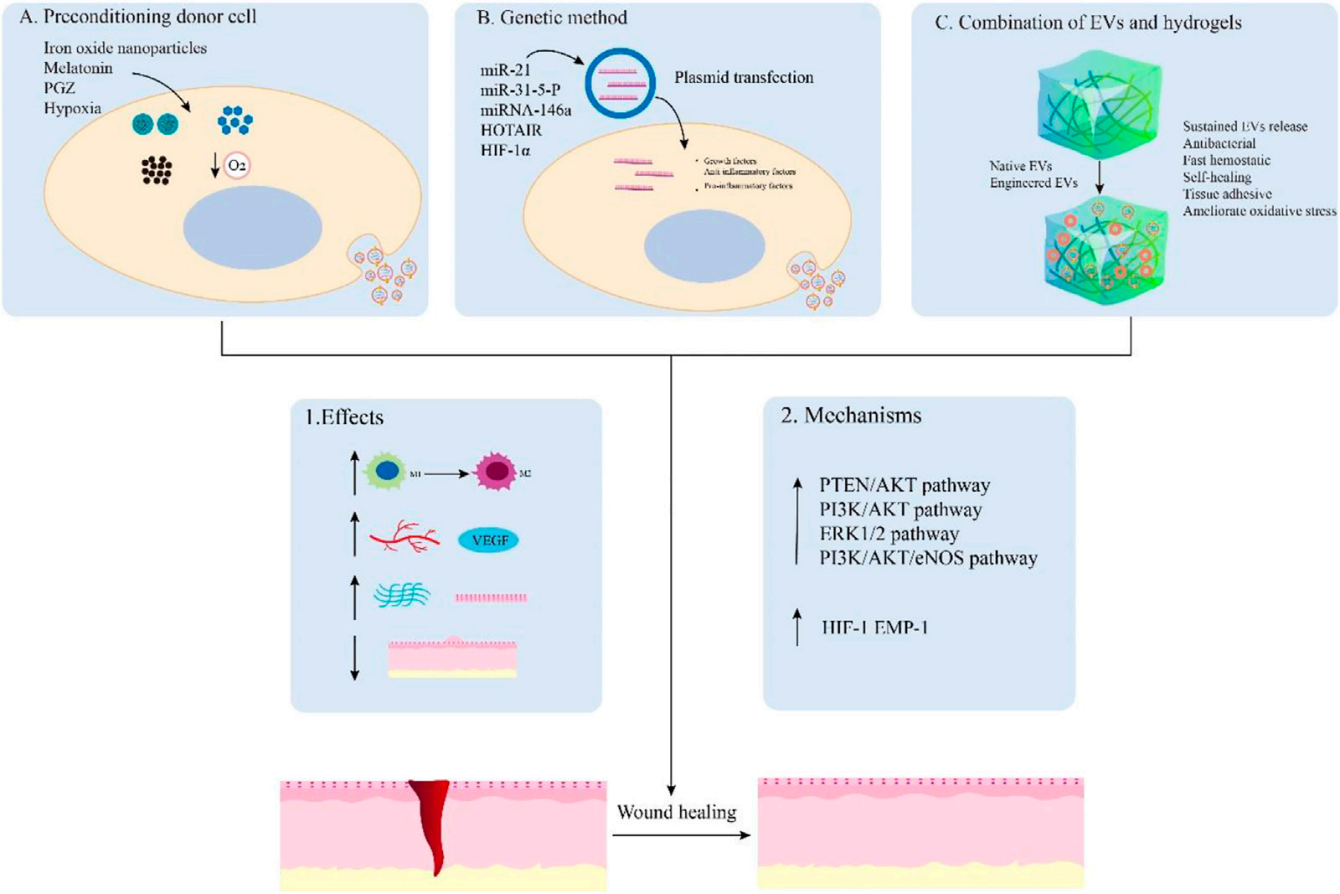
Main examples of engineered EVs for wound healing. EVs can be engineered by preconditioning donor cells **(A)**, genetic method **(B)**, and combination with hydrogels **(C)** to promote wound healing. These engineered EVs can achieve enhanced effects on the regulation of inflammation, angiogenesis, remodeling, and scar formation by activating relevant signaling pathways. Hydrogels and nanomaterials can also synergistically enhance wound healing through sustained EVs release, antibacterial, fast hemostatic, self-healing, tissue adhesive, and improved oxidative stress capabilities.

With the rapid development of EV engineering methodology, a large number of studies have focused on this research field to further improve the application of engineered EVs in wound healing Figure 4 and Table 2

**TABLE 2:** The examples of engineered EVs for wound healing.

EV origin	Model	Disease	Route	Engineering methodology	Effector molecules; pathways	Effects	Ref
Human BMSCs	*In vitro In vivo* (Rat)	Diabetic skin wound	Subcutaneous injection	Preconditioning donor cells with melatonin	IL-1β, TNF-α iNOS, IL-10, and Arg-1; PTEN/AKT pathway	*In vitro*: ↓pro-inflammatory factors ↑anti-inflammatory factors ↑ratio of M2 polarization to M1 polarization *In vivo*: ↓inflammation ↑collagen synthesis ↑angiogenesis	Liu et al. (2020)
BMSCs	*In vitro In vivo* (Rat)	Diabetic skin wound	Subcutaneous injection	Preconditioning donor cells with pioglitazone	VEGF and CD31; PI3K/AKT/eNOS pathway	*In vitro*: ↑HUVECs migration and proliferation ↑HUVECs tube formation ↑VEGF expression *In vivo*: ↑collagen deposition and ECM remodeling ↑VEGF and CD31 expression ↑angiogenesis	Hu et al. (2021)
Human ADSCs	*In vitro In vivo* (Mouse)	Diabetic skin wound	Subcutaneous injection	Preconditioning donor cells with hypoxia conditions	COLI, COLIII, TGF-β, EGF, bFGF, IL-6 and CD31; PI3K/AKT pathway	*In vitro*: ↑fibroblasts proliferation and migration ↑the secretion of extracellular matrix and growth factors ↑ratio of M2 polarization to M1 polarization *In vivo*: ↓inflammatory cytokines ↑re-epithelialization ↑angiogenesis	Wang et al. (2021)
Human BMSCs	*In vitro In vivo* (Rat)	Full-thickness skin wound	Subcutaneous injection	Preconditioning donor cells with MNPs and SMF	MiR-21-5p, and SPRY2; PI3K/AKT and ERK1/2 pathways	*In vitro*: ↑migration and proliferation of HUVECs and HSFs ↑HUVECs tube formation ↑VEGF expression *In vivo*: ↑wound closure ↓scar widths ↑angiogenesis	Wu et al. (2020)
Human ADSCs	*In vitro In vivo* (Mouse)	Full-thickness skin wound	Subcutaneous injection	Overexpressing miR-21	MiR-21, TGF-βI, MMP-2 and TIMP-1; PI3K/AKT signal pathway	*In vitro*: ↑HaCaTs migration and proliferation ↓TGF-βI expression ↑VEGF expression ↑the MMP-9 and TIMP-2 protein expression *In vivo*: ↑wound healing velocity	Yang et al. (2020b)
HEK293	*In vitro In vivo* (Rat)	Diabetic skin wound	Subcutaneous injection	Overexpressing miR-31-5p	MiR-31-5p, HIF-1, EMP-1; HIF pathway	*In vitro*: ↑HaCaTs migration and proliferation ↑ECs migration and proliferation ↑HFF-1 cells migration and proliferation *In vivo*: ↑angiogenesis ↑fibrogenesis ↑re-epithelization	Huang et al. (2021)
BMSCs	*In vitro In vivo* (Mouse)	Diabetic skin wound	Subcutaneous injection	Overexpressing HOTAIR	HOTAIR and VEGF; not studied	*In vitro*: ↑VEGF expression ↑HUVECs and HDMECs proliferation and migration *In vivo*: ↑wound closure ↑new blood vessels	Born et al. (2022)
ASCs	*In vitro In vivo* (Mouse)	Diabetic skin wound	Apply on the wound bed (covered with Tegaderm Film and gaze)	Combined with FEP hydrogel (F127-PEI and APu)	MiR-126, miR-130a, miR-132, miR-let7b and miR-let7c; not studied	*In vitro*: ↑ECs migration and proliferation ↑ECs tube formation *In vivo*: ↑angiogenesis ↑cell proliferation and granulation tissue formation ↑collagen deposition and remodeling ↑re-epithelization ↓scar tissue formation ↑skin appendage regeneration. Other effects of hydrogel: antibacterial activity; fast hemostatic ability; self-healing behavior; tissue-adhesive and good UV-shielding performance	Wang et al. (2019)
Human BMSCs	*In vitro In vivo* (Rat and Rabbit)	Full-thickness skin wound	Apply to the wound surface (covered with a sterile gaze)	Combined with BSSPD hydrogel	MiR-29b-3p; PI3K/Akt, Erk1/2, and Smad3/TGFβ1 pathways	*In vitro*: ↑ECs migration and proliferation ↑fibroblasts migration and proliferation ↑angiogenesis and collagen deposition ↓excessive capillary proliferation and collagen deposition *In vivo*: ↑uniform vascular structure distribution ↑regular collagen arrangement ↓volume of hyperplastic scar tissue ↑skin appendage regeneration	Shen et al. (2021)
M2-Mφs	*In vitro In vivo* (Mouse)	Full-thickness skin wound	Subcutaneous injection (covered with Tegaderm Film)	Combined with PEG hydrogel	MiR-301b-3p, miR-149-5p, miR-125b-5p, miR-26a-5p, and miR-15a-5p; TLR4/NF-κB pathway	*In vitro*: ↑induction of M2-Mφ polarization *In vivo*: ↓acute inflammation ↑induction of M2-Mφ polarizationm ↑efficiency and quality of wound care ↑dermal adipogenesis and hair follicle regeneration	Kwak et al. (2022)
M2-Mφs	*In vitro In vivo* (Mouse)	Diabetic skin wound	Subcutaneous injection (covered with Tegader TM Film)	Overexpressing miRNA-223 and combined with HA@MnO2/FGF-2/Exos hydrogel	MiR-223 FGF-2; not studied	*In vitro*: ↓ROS damage ↑HSFs and HUVECs proliferation ↑HUVECs angiogenesis *In vivo*: ↓inflammation ↑angiogenesis ↑cell proliferation ↑granulation tissue formation ↑re-epithelization ↓ROS damage ↑supply of oxygen Other effects of hydrogel: antibacterial activity; hemostatic ability; self-healing ability; adhesive ability	Xiong et al. (2022)
SMSCs	*In vitro In vivo* (Rat)	Diabetic skin wound	Apply on the wound bed (covered with Tegaderm film)	Overexpressing miR-126-3p and combined with CS hydrogel	MiR-126-3p; AKT and ERK1/2 pathway	*In vitro*: HMEC-1 migration and tube formation *In vivo*: ↑wound closure ↑new blood vessels formation and maturation ↑re-epithelialization ↑mature granulation tissue ↑collagen alignment and deposition ↑the development of hair follicles and sebaceous gland	Tao et al. (2017)

VEGF, vascular endothelial growth factor; MNPs, magnetic nanoparticles; SMF, static magnetic field; HSFs, human skin fibroblasts; HaCaT, human keratinocyte cells; MPP, matrix metalloprotein; TIMP, tissue inhibitor of metalloproteinases; HOTAIR, long non-coding RNA HOX transcript antisense RNA; HDMECs, human dermal microvascular endothelial cells; ASCs, adipose stromal cell; APu, Aldehyde pullulan; F127-PEI , Pluronic F127 grafting polyethylenimine; BSSPD, bilayered thiolated alginate/PEG diacrylate; M2-Mφs, M2 macrophages; PEG, poly (ethylene glycol); ROS, reactive oxygen species; SMSCs, synovium mesenchymal stem cells; CS, chitosan.

### 6.1 EVs derived from preconditioning donor cells for wound healing

Previous studies have shown that preconditioning donor cells is a promising method to improve their biological activity and function in tissue regeneration by various pretreatments including drugs, cytokines, and physical factors (Yang et al., 2016; Hu and Li, 2018). Herein, we describe EVs derived from preconditioning donor cells to exhibit an enhanced ability that promote wound healing. For instance, melatonin-pretreated hBMSCs-derived exosomes (MT-Exos) enhance diabetic wound healing by increasing the macrophage polarization toward the M2 by targeting the PTEN/AKT signaling pathway (Liu et al., 2020). Exosomes released from BMSCs pretreated with pioglitazone (PGZ-Exos) also enhance diabetic wound healing. Since PGZ-Exos improve the angiogenesis ability of HUVECs by activating the PI3K/AKT/eNOS pathway (Hu et al., 2021). Hypoxia leads to a persistent inflammatory environment in diabetic wounds and delay wound healing. Nevertheless, hypoxic ADSCs-derived exosomes (HypADSC-exos) can promote fibroblast proliferation and migration by upregulating the PI3K/Akt pathway, increasing the secretion of vascular growth factors and extracellular matrix, which accelerates wound healing in the diabetic rat model (Wang et al., 2021). In addition, EVs derived from BMSCs stimulated by Fe_3_O_4_ nanoparticles and static magnetic field (mag-BMSC-Exos) can upregulate miR-21-5p to promote angiogenesis and wound healing. The overexpressing miR-21-5p may inhibit SPRY2 and activate PI3K/AKT and ERK1/2 pathways for achieving these effects (Wu et al., 2020). These studies indicate that the EVs obtained from pretreated donor cells are promising in promoting wound healing.

### 6.2 Genetic manipulation of EVs for wound healing

It has been shown that the use of genetically modified engineered EVs is also a novel strategy to promote wound healing. These engineered EVs can be genetically manipulated to obtain desired effects on tissue repair. The overexpressing micoRNA-21 exosomes obtained from ADSCs by plasmid transfection can accelerate wound healing. These engineered exosomes can upregulate the expression of MMP-9 through the PI3K/AKT pathway for promoting the migration and proliferation abilities of the HaCaT cells to achieve this effect (Yang et al., 2020b). EVs from human embryonic kidney 293 cells (HEK293) with miR-31-5p overexpression can enhance angiogenesis, fibrogenesis and reepithelization by inhibiting factor-inhibiting HIF-1 and epithelial membrane protein-1 (EMP-1) for diabetic wound healing (Huang et al., 2021). Moreover, HOTAIR, a long non-coding RNA HOX transcript antisense RNA, is crucial in regulating angiogenic effects. The EVs from BMSCs transfected to overexpress HOTAIR can increase the production of angiogenic protein vascular endothelial growth factor to induce an increased number of blood vessels in wounds, which accelerates chronic wound healing in a diabetic mouse model (Born et al., 2022). Thus, these engineered EVs by genetic modification are effective therapeutic agents for the healing of chronic wounds.

### 6.3 Hydrogels combined with EVs for wound healing

Hydrogels, a type of biomaterial that can form matrices with plenty of water, are used as ideal therapeutic agents for drug delivery systems owing to their cross-linked networks of three-dimensional hydrophilic polymers (Peppas et al., 2006). Various types of hydrogels can be modified to achieve desired functions including moisturizing, adhesiveness, antibacterial, and self-healing abilities. It is important to design a non-invasive, simple, and active system for clinical EVs delivery in disease treatment. As a result, more and more studies focus on hydrogels as EV carriers in regenerative medicine (Akbari et al., 2020).

For instance, exosomes derived from adipose stromal cells are encapsulated in an injectable adhesive thermosensitive multifunctional polysaccharide-based hydrogel (FEP) with the ability to sustain pH-responsive exosome release. The hydrogel has multiple functions including efficient antibacterial activity/multidrug-resistant bacteria, fast hemostatic performance, self-healing characteristic, tissue-adhesive, and good UV-shielding ability. FEP@exosomes hydrogel can significantly lead to faster wound healing by promoting cell proliferation, granulation tissue formation, angiogenesis, collagen deposition, remodeling, and re-epithelialization processes in a diabetic wound model (Wang et al., 2019). Additionally, small extracellular vesicles derived from BMSCs (B-sEVs) were sequentially released by a bilayer thiolated alginate/PEG diacrylate (BSSPD) hydrogel for rapid and scarless wound healing. The lower and upper layers of this hydrogel release B-sEVs and sEVs secreted by miR-29b-3p-enriched BMSCs respectively, resulting in a more uniform vascular structure distribution, more regular collagen arrangement, and smaller hyperplastic scar tissue in a full-thickness skin defect rat model (Shen et al., 2021). Interestingly, based on the ability of exosomes from M2 macrophages to drive M2 macrophage polarization, researchers designed a novel hydrogel that loaded and controlled released exosomes derived from M2 macrophages. This degradable poly (ethylene glycol) (PEG) hydrogel could retain the physicochemical properties of exosomes and release them within 6–27 days, which reduced acute inflammation by inducing M2 macrophage polarization to promote rapid wound closure and increase the efficiency of wound healing (Kwak et al., 2022). Additionally, the hydrogels containing EVs with overexpressing miRNA by genetic modification can achieve effective treatment for wound healing. The novel HA@MnO2/FGF-2/Exos hydrogel contains exosomes derived from M2 macrophages with overexpressing miR-223 enhances diabetic wound healing through multifunctional effects. The HA hydrogel has the ability of self-healing and antibacterial capabilities, while the MnO2/ε-PL nanosheet supplies oxygen to ameliorate oxidative stress by catalyzing H2O2 in the wound area. Meanwhile, the miR-223-overexpressing exosome and FGF-2 promote angiogenesis and epithelization for wound healing. Therefore, the All-in-One hydrogel presents a promising method for chronic diabetic wound healing (Xiong et al., 2022). Similarly, chitosan hydrogel incorporates exosomes derived from miR‐126‐3p‐overexpressing synovium mesenchymal stem cells (SMSCs) to overcome the inability of SMSCs to promote angiogenesis. This hydrogel can enhance diabetic wound repair by inducing the proliferation of dermal fibroblasts and dermal microvascular endothelial cells, stimulating re‐epithelialization, angiogenesis, and collagen maturation *in vitro* and vivo respectively (Tao et al., 2017). To conclude, these findings show that hydrogel combined with EVs has promising clinical application prospects in promoting wound healing.

## 7 Conclusion and perspectives

EVs have excellent prospects in clinical application owing to their strong bioactivity, remarkable function, and high biocompatibility. With the development in the past decade, scientists have made great efforts to explore this field to better understand the biological and functional role of EVs in physiology and pathology (Yates et al., 2022). Compared with cell therapy, the advantage of EV-based treatment is outstanding on an ethical level, isolation, storage, and transportation (Jing et al., 2018). Furthermore, the biocompatible characteristics of EVs reduce the risk of systemic toxicity which is commonly observed in other nanomaterials. Therefore, substantial research progress has been made in the field of EVs isolated from different cell sources to promote wound healing. The therapeutic application of EVs in tissue repair has been explored in numerous preclinical studies and several clinical trials. What’s more, researchers have also focused on applying advanced strategies to design, engineer, and modify EVs to enhance their targeting ability, loading efficiency, and therapeutic efficacy (Fan et al., 2017).

However, some challenges need to be addressed. Since the poor reproducibility and time-consuming procedures of traditional methods such as ultracentrifugation results in a low yield of EVs without high purity, it is difficult to meet the demand for large-scale stable native and engineered EVs in clinical applications (Witwer et al., 2019). Before successfully applying EV products to clinical applications, it is essential that the bioactivity of EV products is reproducible and meet pre-defined quantitative criteria of identity and potency (Gimona et al., 2021). These challenges need to be further explored and addressed per previous scientific publications and guidelines of the International Society for Extracellular Vesicles (ISEV). Furthermore, to merely depict functions of EVs in a crude, potentially contaminated, and heterogeneous preparation, future studies should also report specific information about ascribing a specific function to EVs or subtypes of EVs for the development of good potency assays of EV products (Théry et al., 2018; Gimona et al., 2021). Surprisingly, some researchers used bioreactors and developed streamlined purification protocols *via* microfluidic devices to overcome the difficulties. The hollow-fiber bioreactor for massive production of engineered EVs with more than 40-fold yields than traditional cell culture has been developed (Watson et al., 2016). Moreover, it is vital to standardize preparation and ensure quality control of engineered EVs for their application in therapy.

EVs can be obtained from two sources: autologous or exogenous. Although autologous EVs have the property of immune compatibility, there are several disadvantages including low production yield, an ordeal to standardize, and unpredictable bioactivity limiting their application. By contrast, EVs derived from exogenous sources have higher yields and can be standardized and modified for various clinical applications. Additionally, EVs released from cells have immunogenicity owing to their various bioactive immune molecules, which leads to the host immune system eliminating these EVs through an immune response. Meanwhile, the cargoes and compositions of EVs can be changed during the engineering transformation of EVs, inducing immunogenicity as a result.

Thus, scientists need to explore new approaches to engineering EVs to avoid adverse effects, and corresponding examinations should be performed to prevent potential immune side effects before the clinical application of engineered EVs (Lim et al., 2019).

The study of the effects of tissue-derived EVs (Ti-EVs) in wound healing is also needed in further progress. Ti‐EVs regulate intercellular signal transduction in the extracellular interstitium. Compared with the EVs obtained from body fluids or cell culture supernatants, they have various advantages including accurate reflection of tissue microenvironment, tissue specificity, and minimal contaminants (Camino et al., 2020; Crescitelli et al., 2020). Moreover, a large number of studies have shown that Ti-EVs play a promising role in the treatment of various diseases, such as multiple cancers, Alzheimer’s disease, and metabolic diseases (Li et al., 2021). Nevertheless, there are no relevant studies on Ti-EVs promoting wound healing currently. Consequently, to promote the clinical application of EVs in wound healing better, future research should pay more attention to Ti-EVs and engineered Ti-EVs. Most of the recent papers on EVs promoting wound repair focused on miRNAs as effector molecules. However, studies were showing that most exosomes derived from standard preparations do not harbor many copies of miRNA molecules, which indicated that miRNAs may not be able to elicit a biological response as the frequency of transformation of recipient cells with sufficient configuration and concentration is rare (Chevillet et al., 2014; Albanese et al., 2021). Compared with miRNA, proteins in typical therapeutic MSC-exosome doses have the potential to trigger biologically relevant responses, such as ATP-producing glycolytic enzymes (Toh et al., 2018). Therefore, studying proteins of EVs for promoting wound healing as the main driver is the next research field that scientists need to pay attention to.

The administration route and the dosage regimen are the future research directions that need to be optimized. For the treatment of wounds, the common route of administration of EVs is a subcutaneous injection. However, the process leads to the rapid metabolism and elimination of EVs, which reduces their therapeutic effectiveness. As a result, it is crucial to develop new methods to solve this problem. For example, applying engineered approaches, such as the combination of EVs and hydrogels which slowly and continuously release EVs for prolonging their stay time in the wound. Hydrogels or other biomaterials can also enhance wound healing due to their antibacterial ability, relieving oxidative stress and providing a moisturizing environment. In addition, an increasing number of studies have focused on improving the targeting ability of EVs by engineering strategies to treat diseases including cardiovascular diseases and cancer through intravenous injection of EVs. This administration route may be the next way for EVs to enhance wound healing. A recent study indicated 10^10^–10^11^ EVs to be an effective therapeutic dosage for the treatment of one patient with graft-versus-host disease (Kordelas et al., 2014). The appropriate dosage of EVs in wound healing needs to be further determined.

Overall, both native and engineered EVs can exert promising effects in wound healing by participating in various physiological processes, such as regulating inflammation, inducing angiogenesis, enhancing cell proliferation and migration, synthesizing ECM, and reducing scar formation. Importantly, recent studies on the application of engineered EVs to accelerate wound healing have better effects, suggesting that the field of using engineered EVs as a new method for wound treatment is attractive and promising. Therefore, further work should focus on optimizing and stabilizing these EVs’ engineered strategies and achieving highly scalable production of engineered EVs for future clinical applications.

## References

[B1] Abels E. R. Breakefield X. O. (2016). Introduction to extracellular vesicles: Biogenesis, RNA cargo selection, content, release, and uptake. Cell. Mol. Neurobiol. 36 (3), 301–312. 10.1007/s10571-016-0366-z 27053351 PMC5546313

[B2] Akbari A. Jabbari N. Sharifi R. Ahmadi M. Vahhabi A. Seyedzadeh S. J. (2020). Free and hydrogel encapsulated exosome-based therapies in regenerative medicine. Life Sci. 249, 117447. 10.1016/j.lfs.2020.117447 32087234

[B3] Albanese M. Chen Y. A. Hüls C. Gärtner K. Tagawa T. Mejias-Perez E. (2021). MicroRNAs are minor constituents of extracellular vesicles that are rarely delivered to target cells. PLoS Genet. 17 (12), e1009951. 10.1371/journal.pgen.1009951 34871319 PMC8675925

[B4] An Y. Lin S. Tan X. Zhu S. Nie F. Zhen Y. (2021). Exosomes from adipose-derived stem cells and application to skin wound healing. Cell Prolif. 54 (3), e12993. 10.1111/cpr.12993 33458899 PMC7941238

[B5] Armstrong J. P. Holme M. N. Stevens M. M. (2017). Re-engineering extracellular vesicles as smart nanoscale therapeutics. ACS Nano 11 (1), 69–83. 10.1021/acsnano.6b07607 28068069 PMC5604727

[B6] Baum C. L. Arpey C. J. (2005). Normal cutaneous wound healing: Clinical correlation with cellular and molecular events. Dermatol. Surg. 31 (6), 674–686. 10.1111/j.1524-4725.2005.31612 15996419

[B7] Born L. J. Chang K. H. Shoureshi P. Lay F. Bengali S. Hsu A. T. W. (2022). HOTAIR-loaded mesenchymal stem/stromal cell extracellular vesicles enhance angiogenesis and wound healing. Adv. Healthc. Mat. 11 (5), e2002070. 10.1002/adhm.202002070 PMC8522167 33870645

[B8] Camino T. Lago-Baameiro N. Martis-Sueiro A. Couto I. Santos F. Baltar J. (2020). Deciphering adipose tissue extracellular vesicles protein cargo and its role in obesity. Int. J. Mol. Sci. 21 (24), 9366. 10.3390/ijms21249366 33316953 PMC7764772

[B9] Chargaff E. West R. (1946). The biological significance of the thromboplastic protein of blood. J. Biol. Chem. 166 (1), 189–197. 10.1016/s0021-9258(17)34997-9 20273687

[B10] Chen J. Zhou R. Liang Y. Fu X. Wang D. Wang C. (2019). Blockade of lncRNA-ASLNCS5088-enriched exosome generation in M2 macrophages by GW4869 dampens the effect of M2 macrophages on orchestrating fibroblast activation. FASEB J. 33 (11), 12200–12212. 10.1096/fj.201901610 31373848 PMC6902732

[B11] Chevillet J. R. Kang Q. Ruf I. K. Briggs H. A. Vojtech L. N. Hughes S. M. (2014). Quantitative and stoichiometric analysis of the microRNA content of exosomes. Proc. Natl. Acad. Sci. U. S. A. 111 (41), 14888–14893. 10.1073/pnas.1408301111 25267620 PMC4205618

[B12] Cooper D. R. Wang C. Patel R. Trujillo A. Patel N. A. Prather J. (2018). Human adipose-derived stem cell conditioned media and exosomes containing MALAT1 promote human dermal fibroblast migration and ischemic wound healing. Adv. Wound Care (New. Rochelle. 7 (9), 299–308. 10.1089/wound.2017.0775 30263873 PMC6158770

[B13] Cooper J. M. Wiklander P. B. Nordin J. Z. Al-Shawi R. Wood M. J. Vithlani M. (2014). Systemic exosomal siRNA delivery reduced alpha-synuclein aggregates in brains of transgenic mice. Mov. Disord. 29 (12), 1476–1485. 10.1002/mds.25978 25112864 PMC4204174

[B14] Crescitelli R. Lässer C. Jang S. C. Cvjetkovic A. Malmhäll C. Karimi N. (2020). Subpopulations of extracellular vesicles from human metastatic melanoma tissue identified by quantitative proteomics after optimized isolation. J. Extracell. Vesicles 9 (1), 1722433. 10.1080/20013078.2020.1722433 32128073 PMC7034452

[B15] Doherty G. J. McMahon H. T. (2009). Mechanisms of endocytosis. Annu. Rev. Biochem. 78, 857–902. 10.1146/annurev.biochem.78.081307.110540 19317650

[B16] Eming S. A. Krieg T. Davidson J. M. (2007). Inflammation in wound repair: Molecular and cellular mechanisms. J. Dermatol. 127 (3), 514–525. 10.1038/sj.jid.5700701 17299434

[B17] Fan W. P. Yung B. Huang P. Chen X. Y. (2017). Nanotechnology for multimodal synergistic cancer therapy. Chem. Rev. 117 (22), 13566–13638. 10.1021/acs.chemrev.7b00258 29048884

[B18] Fang S. Xu C. Zhang Y. Xue C. Yang C. Bi H. (2016). Umbilical cord-derived mesenchymal stem cell-derived exosomal MicroRNAs suppress myofibroblast differentiation by inhibiting the transforming growth factor-β/SMAD2 pathway during wound healing. Stem Cells Transl. Med. 5 (10), 1425–1439. 10.5966/sctm.2015-0367 27388239 PMC5031180

[B19] Fuhrmann G. Serio A. Mazo M. Nair R. Stevens M. M. (2015). Active loading into extracellular vesicles significantly improves the cellular uptake and photodynamic effect of porphyrins. J. Control. Release 205, 35–44. 10.1016/j.jconrel.2014.11.029 25483424

[B20] Furie B. Furie B. C. (2008). Mechanisms of thrombus formation. N. Engl. J. Med. Overseas. Ed. 359 (9), 938–949. 10.1056/nejmra0801082 18753650

[B21] Gantwerker E. A. Hom D. B. (2011). Skin: Histology and physiology of wound healing. Facial Plast. Surg. Clin. North Am. 19 (3), 441–453. 10.1016/j.fsc.2011.06.009 21856533

[B22] Géminard C. De Gassart A. Blanc L. Vidal M. (2004). Degradation of AP2 during reticulocyte maturation enhances binding of hsc70 and Alix to a common site on TFR for sorting into exosomes. Traffic 5 (3), 181–193. 10.1111/j.1600-0854.2004.0167.x 15086793

[B23] Gimona M. Brizzi M. F. Choo A. B. H. Dominici M. Davidson S. M. Grillari J. (2021). Critical considerations for the development of potency tests for therapeutic applications of mesenchymal stromal cell-derived small extracellular vesicles. Cytotherapy 23 (5), 373–380. 10.1016/j.jcyt.2021.01.001 33934807

[B24] Glady A. Vandebroek A. Yasui M. (2021). Human keratinocyte-derived extracellular vesicles activate the MAPKinase pathway and promote cell migration and proliferation *in vitro* . Inflamm. Regen. 41 (1), 4. 10.1186/s41232-021-00154-x 33526070 PMC7852286

[B25] Goetzl E. J. Schwartz J. B. Mustapic M. Lobach I. V. Daneman R. Abner E. L. (2017). Altered cargo proteins of human plasma endothelial cell-derived exosomes in atherosclerotic cerebrovascular disease. FASEB J. 31 (8), 3689–3694. 10.1096/fj.201700149 28476896 PMC5503715

[B26] Guillamat-Prats R. (2021). The role of MSC in wound healing, scarring and regeneration. Cells 10 (7), 1729. 10.3390/cells10071729 34359898 PMC8305394

[B27] Guo S. C. Tao S. C. Yin W. J. Qi X. Yuan T. Zhang C. Q. (2017). Exosomes derived from platelet-rich plasma promote the re-epithelization of chronic cutaneous wounds via activation of YAP in a diabetic rat model. Theranostics 7 (1), 81–96. 10.7150/thno.16803 28042318 PMC5196887

[B28] Gusachenko O. N. Zenkova M. A. Vlassov V. V. (2013). Nucleic acids in exosomes: Disease markers and intercellular communication molecules. Biochem. Mosc. 78 (1), 1–7. 10.1134/s000629791301001x 23379554

[B29] Han G. Ceilley R. (2017). Chronic wound healing: A review of current management and treatments. Adv. Ther. 34 (3), 599–610. 10.1007/s12325-017-0478-y 28108895 PMC5350204

[B30] Han X. Wu P. Li L. Sahal H. M. Ji C. Zhang J. (2021). Exosomes derived from autologous dermal fibroblasts promote diabetic cutaneous wound healing through the Akt/β-catenin pathway. Cell Cycle 20 (5-6), 616–629. 10.1080/15384101.2021.1894813 33685347 PMC8018430

[B31] Haney M. J. Klyachko N. L. Zhao Y. Gupta R. Plotnikova E. G. He Z. (2015). Exosomes as drug delivery vehicles for Parkinson's disease therapy. J. Control. Release 207, 18–30. 10.1016/j.jconrel.2015.03.033 25836593 PMC4430381

[B32] Hao Y. Song H. Zhou Z. Chen X. Li H. Zhang Y. (2021). Promotion or inhibition of extracellular vesicle release: Emerging therapeutic opportunities. J. Control. Release 340, 136–148. 10.1016/j.jconrel.2021.10.019 34695524

[B33] Harp D. Driss A. Mehrabi S. Chowdhury I. Xu W. Liu D. (2016). Exosomes derived from endometriotic stromal cells have enhanced angiogenic effects *in vitro* . Cell Tissue Res. 365 (1), 187–196. 10.1007/s00441-016-2358-1 26841879 PMC4917586

[B34] Hart J. (2002). Inflammation. 2: Its role in the healing of chronic wounds. J. Wound Care 11 (7), 245–249. 10.12968/jowc.2002.11.7.26416 12192842

[B35] Hu C. Li L. (2018). Preconditioning influences mesenchymal stem cell properties *in vitro* and *in vivo* . J. Cell. Mol. Med. 22 (3), 1428–1442. 10.1111/jcmm.13492 29392844 PMC5824372

[B36] Hu L. Wang J. Zhou X. Xiong Z. Zhao J. Yu R. (2016). Exosomes derived from human adipose mensenchymal stem cells accelerates cutaneous wound healing via optimizing the characteristics of fibroblasts. Sci. Rep. 6, 32993. 10.1038/srep32993 27615560 PMC5018733

[B37] Hu Y. Rao S. S. Wang Z. X. Cao J. Tan Y. J. Luo J. (2018). Exosomes from human umbilical cord blood accelerate cutaneous wound healing through miR-21-3p-mediated promotion of angiogenesis and fibroblast function. Theranostics 8 (1), 169–184. 10.7150/thno.21234 29290800 PMC5743467

[B38] Hu Y. Tao R. Chen L. Xiong Y. Xue H. Hu L. (2021). Exosomes derived from pioglitazone-pretreated MSCs accelerate diabetic wound healing through enhancing angiogenesis. J. Nanobiotechnology 19 (1), 150. 10.1186/s12951-021-00894-5 34020670 PMC8139165

[B39] Huang J. Yu M. Yin W. Liang B. Li A. Li J. (2021). Development of a novel RNAi therapy: Engineered miR-31 exosomes promoted the healing of diabetic wounds. Bioact. Mat. 6 (9), 2841–2853. 10.1016/j.bioactmat.2021.02.007 PMC7905076 33718666

[B40] Huang P. Bi J. Owen G. R. Chen W. Rokka A. Koivisto L. (2015). Keratinocyte microvesicles regulate the expression of multiple genes in dermal fibroblasts. J. Dermatol. 135 (12), 3051–3059. 10.1038/jid.2015.320 26288358

[B41] Jahn R. Südhof T. C. (1999). Membrane fusion and exocytosis. Annu. Rev. Biochem. 68, 863–911. 10.1146/annurev.biochem.68.1.863 10872468

[B42] Jang S. C. Kim O. Y. Yoon C. M. Choi D. S. Roh T. Y. Park J. (2013). Bioinspired exosome-mimetic nanovesicles for targeted delivery of chemotherapeutics to malignant tumors. ACS Nano 7 (9), 7698–7710. 10.1021/nn402232g 24004438

[B43] Jia G. Han Y. An Y. Ding Y. He C. Wang X. (2018). NRP-1 targeted and cargo-loaded exosomes facilitate simultaneous imaging and therapy of glioma *in vitro* and *in vivo* . Biomaterials 178, 302–316. 10.1016/j.biomaterials.2018.06.029 29982104

[B44] Jing H. He X. Zheng J. (2018). Exosomes and regenerative medicine: State of the art and perspectives. Transl. Res. 196, 1–16. 10.1016/j.trsl.2018.01.005 29432720

[B45] Kahlert C. Kalluri R. (2013). Exosomes in tumor microenvironment influence cancer progression and metastasis. J. Mol. Med. 91 (4), 431–437. 10.1007/s00109-013-1020-6 23519402 PMC4073669

[B46] Kang T. Jones T. M. Naddell C. Bacanamwo M. Calvert J. W. Thompson W. E. (2016). Adipose-derived stem cells induce angiogenesis via microvesicle transport of miRNA-31. Stem Cells Transl. Med. 5 (4), 440–450. 10.5966/sctm.2015-0177 26933040 PMC4798737

[B47] Kirchhausen T. (2000). Clathrin. Annu. Rev. Biochem. 69, 699–727. 10.1146/annurev.biochem.69.1.699 10966473

[B48] Koh T. J. DiPietro L. A. (2011). Inflammation and wound healing: The role of the macrophage. Expert Rev. Mol. Med. 13, e23. 10.1017/s1462399411001943 21740602 PMC3596046

[B49] Kooijmans S. A. A. Fliervoet L. A. L. van der Meel R. Fens M. Heijnen H. F. G. van Bergen En Henegouwen P. M. P. (2016). PEGylated and targeted extracellular vesicles display enhanced cell specificity and circulation time. J. Control. Release 224, 77–85. 10.1016/j.jconrel.2016.01.009 26773767

[B50] Kordelas L. Rebmann V. Ludwig A. K. Radtke S. Ruesing J. Doeppner T. R. (2014). MSC-Derived exosomes: A novel tool to treat therapy-refractory graft-versus-host disease. Leukemia 28 (4), 970–973. 10.1038/leu.2014.41 24445866

[B51] Kwak G. Cheng J. Kim H. Song S. Lee S. J. Yang Y. (2022). Sustained exosome-guided macrophage polarization using hydrolytically degradable PEG hydrogels for cutaneous wound healing: Identification of key proteins and MiRNAs, and sustained release formulation. Small 18 (15), e2200060. 10.1002/smll.202200060 35229462

[B52] Le Blanc K. Mougiakakos D. (2012). Multipotent mesenchymal stromal cells and the innate immune system. Nat. Rev. Immunol. 12 (5), 383–396. 10.1038/nri3209 22531326

[B53] Li M. Wang T. Tian H. Wei G. Zhao L. Shi Y. (2019). Macrophage-derived exosomes accelerate wound healing through their anti-inflammation effects in a diabetic rat model. Artif. Cells Nanomed. Biotechnol. 47 (1), 3793–3803. 10.1080/21691401.2019.1669617 31556314

[B54] Li Q. Zhao H. Chen W. Huang P. Bi J. (2019). Human keratinocyte-derived microvesicle miRNA-21 promotes skin wound healing in diabetic rats through facilitating fibroblast function and angiogenesis. Int. J. Biochem. Cell Biol. 114, 105570. 10.1016/j.biocel.2019.105570 31302227

[B55] Li S. R. Man Q. W. Gao X. Lin H. Wang J. Su F. C. (2021). Tissue-derived extracellular vesicles in cancers and non-cancer diseases: Present and future. J. Extracell. Vesicles 10 (14), e12175. 10.1002/jev2.12175 34918479 PMC8678102

[B56] Li X. Jiang C. Zhao J. (2016). Human endothelial progenitor cells-derived exosomes accelerate cutaneous wound healing in diabetic rats by promoting endothelial function. J. Diabetes Complicat. 30 (6), 986–992. 10.1016/j.jdiacomp.2016.05.009 27236748

[B57] Li X. Liu L. Yang J. Yu Y. Chai J. Wang L. (2016). Exosome derived from human umbilical cord mesenchymal stem cell mediates MiR-181c attenuating burn-induced excessive inflammation. EBioMedicine 8, 72–82. 10.1016/j.ebiom.2016.04.030 27428420 PMC4919539

[B58] Liang G. Kan S. Zhu Y. Feng S. Feng W. Gao S. (2018). Engineered exosome-mediated delivery of functionally active miR-26a and its enhanced suppression effect in HepG2 cells. Int. J. Nanomedicine 13, 585–599. 10.2147/ijn.s154458 29430178 PMC5796471

[B59] Liang X. Zhang L. Wang S. Han Q. Zhao R. C. (2016). Exosomes secreted by mesenchymal stem cells promote endothelial cell angiogenesis by transferring miR-125a. J. Cell Sci. 129 (11), 2182–2189. 10.1242/jcs.170373 27252357

[B60] Lim S. Park J. Shim M. K. Um W. Yoon H. Y. Ryu J. H. (2019). Recent advances and challenges of repurposing nanoparticle-based drug delivery systems to enhance cancer immunotherapy. Theranostics 9 (25), 7906–7923. 10.7150/thno.38425 31695807 PMC6831456

[B61] Liu C. Gao H. Lv P. Liu J. Liu G. (2017). Extracellular vesicles as an efficient nanoplatform for the delivery of therapeutics. Hum. Vaccin. Immunother. 13 (11), 2678–2687. 10.1080/21645515.2017.1363935 28949786 PMC5703411

[B62] Liu W. Yu M. Xie D. Wang L. Ye C. Zhu Q. (2020). Melatonin-stimulated MSC-derived exosomes improve diabetic wound healing through regulating macrophage M1 and M2 polarization by targeting the PTEN/AKT pathway. Stem Cell Res. Ther. 11 (1), 259. 10.1186/s13287-020-01756-x 32600435 PMC7322868

[B63] Lo Sicco C. Reverberi D. Balbi C. Ulivi V. Principi E. Pascucci L. (2017). Mesenchymal stem cell-derived extracellular vesicles as mediators of anti-inflammatory effects: Endorsement of macrophage polarization. Stem Cells Transl. Med. 6 (3), 1018–1028. 10.1002/sctm.16-0363 28186708 PMC5442783

[B64] Martin P. (1997). Wound healing--aiming for perfect skin regeneration. Science 276 (5309), 75–81. 10.1126/science.276.5309.75 9082989

[B65] McBride J. D. Rodriguez-Menocal L. Guzman W. Candanedo A. Garcia-Contreras M. Badiavas E. V. (2017). Bone marrow mesenchymal stem cell-derived CD63(+) exosomes transport Wnt3a exteriorly and enhance dermal fibroblast proliferation, migration, and angiogenesis *in vitro* . Stem Cells Dev. 26 (19), 1384–1398. 10.1089/scd.2017.0087 28679315

[B66] Mentkowski K. I. Snitzer J. D. Rusnak S. Lang J. K. (2018). Therapeutic potential of engineered extracellular vesicles. AAPS J. 20 (3), 50. 10.1208/s12248-018-0211-z 29546642 PMC8299397

[B67] Monguió-Tortajada M. Roura S. Gálvez-Montón C. Pujal J. M. Aran G. Sanjurjo L. (2017). Nanosized UCMSC-derived extracellular vesicles but not conditioned medium exclusively inhibit the inflammatory response of stimulated T cells: Implications for nanomedicine. Theranostics 7 (2), 270–284. 10.7150/thno.16154 28042333 PMC5197063

[B68] Müller U. (2020). Exosome-mediated protection of auditory hair cells from ototoxic insults. J. Clin. 130 (5), 2206–2208. 10.1172/jci135710 PMC7190972 32310224

[B69] Nabi I. R. Le P. U. (2003). Caveolae/raft-dependent endocytosis. J. Cell Biol. 161 (4), 673–677. 10.1083/jcb.200302028 12771123 PMC2199359

[B70] Nakase I. Futaki S. (2015). Combined treatment with a pH-sensitive fusogenic peptide and cationic lipids achieves enhanced cytosolic delivery of exosomes. Sci. Rep. 5, 10112. 10.1038/srep10112 26011176 PMC4443764

[B71] Nawaz M. Fatima F. Vallabhaneni K. C. Penfornis P. Valadi H. Ekström K. (2016). Extracellular vesicles: Evolving factors in stem cell biology. Stem Cells Int. 2016, 1–17. 10.1155/2016/1073140 PMC4663346 26649044

[B72] Nawaz M. Shah N. Zanetti B. R. Maugeri M. Silvestre R. N. Fatima F. (2018). Extracellular vesicles and matrix remodeling enzymes: The emerging roles in extracellular matrix remodeling, progression of diseases and tissue repair. Cells 7 (10), 167. 10.3390/cells7100167 30322133 PMC6210724

[B73] Nosbaum A. Prevel N. Truong H. A. Mehta P. Ettinger M. Scharschmidt T. C. (2016). Cutting edge: Regulatory T cells facilitate cutaneous wound healing. J. I. 196 (5), 2010–2014. 10.4049/jimmunol.1502139 PMC4761457 26826250

[B74] Oh E. J. Gangadaran P. Rajendran R. L. Kim H. M. Oh J. M. Choi K. Y. (2021). Extracellular vesicles derived from fibroblasts promote wound healing by optimizing fibroblast and endothelial cellular functions. Stem Cells 39 (3), 266–279. 10.1002/stem.3310 33289943

[B75] Pascucci L. Coccè V. Bonomi A. Ami D. Ceccarelli P. Ciusani E. (2014). Paclitaxel is incorporated by mesenchymal stromal cells and released in exosomes that inhibit *in vitro* tumor growth: A new approach for drug delivery. J. Control. Release 192, 262–270. 10.1016/j.jconrel.2014.07.042 25084218

[B76] Pegtel D. M. Gould S. J. (2019). Exosomes. Annu. Rev. Biochem. 88, 487–514. 10.1146/annurev-biochem-013118-111902 31220978

[B77] Peppas N. A. Hilt J. Z. Khademhosseini A. Langer R. (2006). Hydrogels in biology and medicine: From molecular principles to bionanotechnology. Adv. Mat. 18 (11), 1345–1360. 10.1002/adma.200501612

[B78] Pittenger M. F. Mackay A. M. Beck S. C. Jaiswal R. K. Douglas R. Mosca J. D. (1999). Multilineage potential of adult human mesenchymal stem cells. Science 284 (5411), 143–147. 10.1126/science.284.5411.143 10102814

[B79] Presolski S. I. Hong V. P. Finn M. G. (2011). Copper-catalyzed azide-alkyne click chemistry for bioconjugation. Curr. Protoc. Chem. Biol. 3 (4), 153–162. 10.1002/9780470559277.ch110148 22844652 PMC3404492

[B80] Qi H. Liu C. Long L. Ren Y. Zhang S. Chang X. (2016). Blood exosomes endowed with magnetic and targeting properties for cancer therapy. ACS Nano 10 (3), 3323–3333. 10.1021/acsnano.5b06939 26938862

[B81] Qi L. Lu Y. Wang Z. Zhang G. (2021). microRNA-106b derived from endothelial cell-secreted extracellular vesicles prevents skin wound healing by inhibiting JMJD3 and RIPK3. J. Cell. Mol. Med. 25 (10), 4551–4561. 10.1111/jcmm.16037 33734576 PMC8107101

[B82] Raiborg C. Stenmark H. (2009). The ESCRT machinery in endosomal sorting of ubiquitylated membrane proteins. Nature 458 (7237), 445–452. 10.1038/nature07961 19325624

[B83] Rani S. Ritter T. (2016). The exosome - a naturally secreted nanoparticle and its application to wound healing. Adv. Mat. 28 (27), 5542–5552. 10.1002/adma.201504009 26678528

[B84] Raposo G. Stoorvogel W. (2013). Extracellular vesicles: Exosomes, microvesicles, and friends. J. Cell Biol. 200 (4), 373–383. 10.1083/jcb.201211138 23420871 PMC3575529

[B85] Ren S. Chen J. Duscher D. Liu Y. Guo G. Kang Y. (2019). Microvesicles from human adipose stem cells promote wound healing by optimizing cellular functions via AKT and ERK signaling pathways. Stem Cell Res. Ther. 10 (1), 47. 10.1186/s13287-019-1152-x 30704535 PMC6357421

[B86] Ridder K. Sevko A. Heide J. Dams M. Rupp A. K. Macas J. (2015). Extracellular vesicle-mediated transfer of functional RNA in the tumor microenvironment. Oncoimmunology 4 (6), e1008371. 10.1080/2162402x.2015.1008371 26155418 PMC4485784

[B87] Robbins P. D. Morelli A. E. (2014). Regulation of immune responses by extracellular vesicles. Nat. Rev. Immunol. 14 (3), 195–208. 10.1038/nri3622 24566916 PMC4350779

[B88] Rodriguez-Menocal L. Shareef S. Salgado M. Shabbir A. Van Badiavas E. (2015). Role of whole bone marrow, whole bone marrow cultured cells, and mesenchymal stem cells in chronic wound healing. Stem Cell Res. Ther. 6, 24. 10.1186/s13287-015-0001-9 25881077 PMC4414366

[B89] Rousselle P. Braye F. Dayan G. (2019). Re-epithelialization of adult skin wounds: Cellular mechanisms and therapeutic strategies. Adv. Drug Deliv. Rev. 146, 344–365. 10.1016/j.addr.2018.06.019 29981800

[B90] Ruiz-Martinez M. Navarro A. Marrades R. M. Viñolas N. Santasusagna S. Muñoz C. (2016). YKT6 expression, exosome release, and survival in non-small cell lung cancer. Oncotarget 7 (32), 51515–51524. 10.18632/oncotarget.9862 27285987 PMC5239493

[B91] Shen Y. Xu G. Huang H. Wang K. Wang H. Lang M. (2021). Sequential release of small extracellular vesicles from bilayered thiolated alginate/polyethylene glycol diacrylate hydrogels for scarless wound healing. ACS Nano 15 (4), 6352–6368. 10.1021/acsnano.0c07714 33723994

[B92] Singer A. J. Clark R. A. (1999). Cutaneous wound healing. N. Engl. J. Med. Overseas. Ed. 341 (10), 738–746. 10.1056/nejm199909023411006 10471461

[B93] Skotland T. Hessvik N. P. Sandvig K. Llorente A. (2019). Exosomal lipid composition and the role of ether lipids and phosphoinositides in exosome biology. J. Lipid Res. 60 (1), 9–18. 10.1194/jlr.r084343 30076207 PMC6314266

[B94] Smyth T. Kullberg M. Malik N. Smith-Jones P. Graner M. W. Anchordoquy T. J. (2015). Biodistribution and delivery efficiency of unmodified tumor-derived exosomes. J. Control. Release 199, 145–155. 10.1016/j.jconrel.2014.12.013 25523519 PMC4441346

[B95] Susa F. Limongi T. Dumontel B. Vighetto V. Cauda V. (2019). Engineered extracellular vesicles as a reliable tool in cancer nanomedicine. Cancers (Basel) 11 (12), 1979. 10.3390/cancers11121979 31835327 PMC6966613

[B96] Tang K. Zhang Y. Zhang H. Xu P. Liu J. Ma J. (2012). Delivery of chemotherapeutic drugs in tumour cell-derived microparticles. Nat. Commun. 3, 1282. 10.1038/ncomms2282 23250412

[B97] Tao S. C. Guo S. C. Li M. Ke Q. F. Guo Y. P. Zhang C. Q. (2017). Chitosan wound dressings incorporating exosomes derived from MicroRNA-126-overexpressing synovium mesenchymal stem cells provide sustained release of exosomes and heal full-thickness skin defects in a diabetic rat model. Stem Cells Transl. Med. 6 (3), 736–747. 10.5966/sctm.2016-0275 28297576 PMC5442792

[B98] Théry C. Witwer K. W. Aikawa E. Alcaraz M. J. Anderson J. D. Andriantsitohaina R. (2018). Clinical research using extracellular vesicles: Insights from the international society for extracellular vesicles 2018 annual meeting. J. Extracell. Vesicles 7 (1), 1535744. 10.1080/20013078.2018.1535744 31162489 PMC6211232

[B99] Ti D. Hao H. Fu X. Han W. (2016). Mesenchymal stem cells-derived exosomal microRNAs contribute to wound inflammation. Sci. China Life Sci. 59 (12), 1305–1312. 10.1007/s11427-016-0240-4 27864711

[B100] Ti D. Hao H. Tong C. Liu J. Dong L. Zheng J. (2015). LPS-preconditioned mesenchymal stromal cells modify macrophage polarization for resolution of chronic inflammation via exosome-shuttled let-7b. J. Transl. Med. 13, 308. 10.1186/s12967-015-0642-6 26386558 PMC4575470

[B101] Tian T. Zhang H. X. He C. P. Fan S. Zhu Y. L. Qi C. (2018). Surface functionalized exosomes as targeted drug delivery vehicles for cerebral ischemia therapy. Biomaterials 150, 137–149. 10.1016/j.biomaterials.2017.10.012 29040874

[B102] Tian Y. Li S. Song J. Ji T. Zhu M. Anderson G. J. (2014). A doxorubicin delivery platform using engineered natural membrane vesicle exosomes for targeted tumor therapy. Biomaterials 35 (7), 2383–2390. 10.1016/j.biomaterials.2013.11.083 24345736

[B103] Toh W. S. Lai R. C. Zhang B. Lim S. K. (2018). MSC exosome works through a protein-based mechanism of action. Biochem. Soc. Trans. 46 (4), 843–853. 10.1042/bst20180079 29986939 PMC6103455

[B104] Vader P. Mol E. A. Pasterkamp G. Schiffelers R. M. (2016). Extracellular vesicles for drug delivery. Adv. Drug Deliv. Rev. 106, 148–156. 10.1016/j.addr.2016.02.006 26928656

[B105] van Dongen H. M. Masoumi N. Witwer K. W. Pegtel D. M. (2016). Extracellular vesicles exploit viral entry routes for cargo delivery. Microbiol. Mol. Biol. Rev. 80 (2), 369–386. 10.1128/mmbr.00063-15 26935137 PMC4867369

[B106] van Niel G. D'Angelo G. Raposo G. (2018). Shedding light on the cell biology of extracellular vesicles. Nat. Rev. Mol. Cell Biol. 19 (4), 213–228. 10.1038/nrm.2017.125 29339798

[B107] Velnar T. Bailey T. Smrkolj V. (2009). The wound healing process: An overview of the cellular and molecular mechanisms. J. Int. Med. Res. 37 (5), 1528–1542. 10.1177/147323000903700531 19930861

[B108] Wang J. Wu H. Peng Y. Zhao Y. Qin Y. Zhang Y. (2021). Hypoxia adipose stem cell-derived exosomes promote high-quality healing of diabetic wound involves activation of PI3K/Akt pathways. J. Nanobiotechnology 19 (1), 202. 10.1186/s12951-021-00942-0 34233694 PMC8261989

[B109] Wang L. Hu L. Zhou X. Xiong Z. Zhang C. Shehada H. M. A. (2017). Exosomes secreted by human adipose mesenchymal stem cells promote scarless cutaneous repair by regulating extracellular matrix remodelling. Sci. Rep. 7 (1), 13321. 10.1038/s41598-017-12919-x 29042658 PMC5645460

[B110] Wang M. Altinoglu S. Takeda Y. S. Xu Q. (2015). Integrating protein engineering and bioorthogonal click conjugation for extracellular vesicle modulation and intracellular delivery. PLoS One 10 (11), e0141860. 10.1371/journal.pone.0141860 26529317 PMC4631329

[B111] Wang M. Wang C. Chen M. Xi Y. Cheng W. Mao C. (2019). Efficient angiogenesis-based diabetic wound healing/skin reconstruction through bioactive antibacterial adhesive ultraviolet shielding nanodressing with exosome release. ACS Nano 13 (9), 10279–10293. 10.1021/acsnano.9b03656 31483606

[B112] Watson D. C. Bayik D. Srivatsan A. Bergamaschi C. Valentin A. Niu G. (2016). Efficient production and enhanced tumor delivery of engineered extracellular vesicles. Biomaterials 105, 195–205. 10.1016/j.biomaterials.2016.07.003 27522254 PMC7156278

[B113] Wei F. Wang A. Wang Q. Han W. Rong R. Wang L. (2020). Plasma endothelial cells-derived extracellular vesicles promote wound healing in diabetes through YAP and the PI3K/Akt/mTOR pathway. Aging (Albany NY) 12 (12), 12002–12018. 10.18632/aging.103366 32570219 PMC7343472

[B114] Witwer K. W. Van Balkom B. W. M. Bruno S. Choo A. Dominici M. Gimona M. (2019). Defining mesenchymal stromal cell (MSC)-derived small extracellular vesicles for therapeutic applications. J. Extracell. Vesicles 8 (1), 1609206. 10.1080/20013078.2019.1609206 31069028 PMC6493293

[B115] Wu D. Kang L. Tian J. Wu Y. Liu J. Li Z. (2020). <p&gt;Exosomes Derived from Bone Mesenchymal Stem Cells with the Stimulation of Fe&lt;sub&gt;3&lt;/sub&gt;O&lt;sub&gt;4&lt;/sub&gt; Nanoparticles and Static Magnetic Field Enhance Wound Healing through Upregulated miR-21-5p&lt;/p&gt;. Int. J. Nanomedicine 15, 7979–7993. 10.2147/ijn.s275650 33116513 PMC7585514

[B116] Xiong Y. Chen L. Liu P. Yu T. Lin C. Yan C. (2022). All-in-One: Multifunctional hydrogel accelerates oxidative diabetic wound healing through timed-release of exosome and fibroblast growth factor. Small 18 (1), e2104229. 10.1002/smll.202104229 34791802

[B117] Xu Y. Lin Z. He L. Qu Y. Ouyang L. Han Y. (2021). Platelet-rich plasma-derived exosomal USP15 promotes cutaneous wound healing via deubiquitinating EIF4A1. Oxid. Med. Cell. Longev. 2021, 1–14. 10.1155/2021/9674809 PMC8371654 34422211

[B118] Yang C. Luo L. Bai X. Shen K. Liu K. Wang J. (2020). Highly-expressed micoRNA-21 in adipose derived stem cell exosomes can enhance the migration and proliferation of the HaCaT cells by increasing the MMP-9 expression through the PI3K/AKT pathway. Arch. Biochem. Biophys. 681, 108259. 10.1016/j.abb.2020.108259 31926164

[B119] Yang J. Chen Z. Pan D. Li H. Shen J. (2020). <p&gt;Umbilical cord-derived mesenchymal stem cell-derived exosomes combined pluronic F127 hydrogel promote chronic diabetic wound healing and complete skin regeneration</p&gt;. Int. J. Nanomedicine 15, 5911–5926. 10.2147/ijn.s249129 32848396 PMC7429232

[B120] Yang J. Liu X. X. Fan H. Tang Q. Shou Z. X. Zuo D. M. (2015). Extracellular vesicles derived from bone marrow mesenchymal stem cells protect against experimental colitis via attenuating colon inflammation, oxidative stress and apoptosis. PLoS One 10 (10), e0140551. 10.1371/journal.pone.0140551 26469068 PMC4607447

[B121] Yang T. Martin P. Fogarty B. Brown A. Schurman K. Phipps R. (2015). Exosome delivered anticancer drugs across the blood-brain barrier for brain cancer therapy in *Danio rerio* . Pharm. Res. 32 (6), 2003–2014. 10.1007/s11095-014-1593-y 25609010 PMC4520542

[B122] Yang Y. Choi H. Seon M. Cho D. Bang S. I. (2016). LL-37 stimulates the functions of adipose-derived stromal/stem cells via early growth response 1 and the MAPK pathway. Stem Cell Res. Ther. 7 (1), 58. 10.1186/s13287-016-0313-4 27095351 PMC4837546

[B123] Yates A. G. Pink R. C. Erdbrügger U. Siljander P. R. Dellar E. R. Pantazi P. (2022). In sickness and in health: The functional role of extracellular vesicles in physiology and pathology *in vivo*: Part II: Pathology: Part II: Pathology. J. Extracell. Vesicles 11 (1), e12190. 10.1002/jev2.12190 35041301 PMC8765328

[B124] Yu B. Shao H. Su C. Jiang Y. Chen X. Bai L. (2016). Exosomes derived from MSCs ameliorate retinal laser injury partially by inhibition of MCP-1. Sci. Rep. 6, 34562. 10.1038/srep34562 27686625 PMC5043341

[B125] Zeng T. Wang X. Wang W. Feng Q. Lao G. Liang Y. (2019). Endothelial cell-derived small extracellular vesicles suppress cutaneous wound healing through regulating fibroblasts autophagy. Clin. Sci. 133 (9), CS20190008. 10.1042/cs20190008 30988132

[B126] Zhang B. Shi Y. Gong A. Pan Z. Shi H. Yang H. (2016). HucMSC exosome-delivered 14-3-3ζ orchestrates self-control of the Wnt response via modulation of YAP during cutaneous regeneration. Stem Cells 34 (10), 2485–2500. 10.1002/stem.2432 27334574

[B127] Zhang B. Wang M. Gong A. Zhang X. Wu X. Zhu Y. (2015). HucMSC-exosome mediated-wnt4 signaling is required for cutaneous wound healing. Stem Cells 33 (7), 2158–2168. 10.1002/stem.1771 24964196

[B128] Zhang D. Lee H. Wang X. Rai A. Groot M. Jin Y. (2018). Exosome-mediated small RNA delivery: A novel therapeutic approach for inflammatory lung responses. Mol. Ther. 26 (9), 2119–2130. 10.1016/j.ymthe.2018.06.007 30005869 PMC6127502

[B129] Zhang J. Chen C. Hu B. Niu X. Liu X. Zhang G. (2016). Exosomes derived from human endothelial progenitor cells accelerate cutaneous wound healing by promoting angiogenesis through erk1/2 signaling. Int. J. Biol. Sci. 12 (12), 1472–1487. 10.7150/ijbs.15514 27994512 PMC5166489

[B130] Zhang J. Guan J. Niu X. Hu G. Guo S. Li Q. (2015). Exosomes released from human induced pluripotent stem cells-derived MSCs facilitate cutaneous wound healing by promoting collagen synthesis and angiogenesis. J. Transl. Med. 13, 49. 10.1186/s12967-015-0417-0 25638205 PMC4371881

[B131] Zhang K. L. Wang Y. J. Sun J. Zhou J. Xing C. Huang G. (2019). Artificial chimeric exosomes for anti-phagocytosis and targeted cancer therapy. Chem. Sci. 10 (5), 1555–1561. 10.1039/c8sc03224f 30809374 PMC6357862

[B132] Zhang Y. Bai X. Shen K. Luo L. Zhao M. Xu C. (2022). Exosomes derived from adipose mesenchymal stem cells promote diabetic chronic wound healing through SIRT3/SOD2. Cells 11 (16), 2568. 10.3390/cells11162568 36010644 PMC9406299

[B133] Zhang Y. Liu Y. Liu H. Tang W. H. (2019). Exosomes: Biogenesis, biologic function and clinical potential. Cell Biosci. 9, 19. 10.1186/s13578-019-0282-2 30815248 PMC6377728

[B134] Zhao B. Zhang Y. Han S. Zhang W. Zhou Q. Guan H. (2017). Exosomes derived from human amniotic epithelial cells accelerate wound healing and inhibit scar formation. J. Mol. Histol. 48 (2), 121–132. 10.1007/s10735-017-9711-x 28229263

[B135] Zhuang X. Xiang X. Grizzle W. Sun D. Zhang S. Axtell R. C. (2011). Treatment of brain inflammatory diseases by delivering exosome encapsulated anti-inflammatory drugs from the nasal region to the brain. Mol. Ther. 19 (10), 1769–1779. 10.1038/mt.2011.164 21915101 PMC3188748

